# Metabolomics differences between silkworms (*Bombyx mori*) reared on fresh mulberry (*Morus*) leaves or artificial diets

**DOI:** 10.1038/s41598-017-11592-4

**Published:** 2017-09-08

**Authors:** Hui-Ling Dong, Sheng-Xiang Zhang, Hui Tao, Zhuo-Hua Chen, Xue Li, Jian-Feng Qiu, Wen-Zhao Cui, Yang-Hu Sima, Wei-Zheng Cui, Shi-Qing Xu

**Affiliations:** 1 School of Biology and Basic Medical Sciences, Medical College, Soochow University, Suzhou, 215123 China; 2 Institute of Agricultural Biotechnology & Ecology (IABE), Soochow University, Suzhou, 215123 China; 3 College of Forestry, Shandong Agricultural University, Taian Shandong, 271018 China; 4 National Engineering Laboratory for Modern Silk (NEAER), Soochow University, Suzhou, 215123 China

## Abstract

Silkworms (*Bombyx mori*) reared on artificial diets have great potential applications in sericulture. However, the mechanisms underlying the enhancement of metabolic utilization by altering silkworm nutrition are unclear. The aim of this study was to investigate the mechanisms responsible for the poor development and low silk protein synthesis efficiency of silkworms fed artificial diets. After multi-generational selection of the ingestive behavior of silkworms to artificial diets, we obtained two strains, one of which developed well and another in which almost all its larvae starved to death on the artificial diets. Subsequently, we analyzed the metabolomics of larval hemolymph by gas chromatography/liquid chromatography–mass spectrometry, and the results showed that vitamins were in critically short supply, whereas the nitrogen metabolic end product of urea and uric acid were enriched substantially, in the hemolymph of the silkworms reared on the artificial diets. Meanwhile, amino acid metabolic disorders, as well as downregulation of carbohydrate metabolism, energy metabolism, and lipid metabolism, co-occurred. Furthermore, 10 male-dominant metabolites and 27 diet-related metabolites that differed between male and female silkworms were identified. These findings provide important insights into the regulation of silkworm metabolism and silk protein synthesis when silkworms adapt to an artificial diet.

## Introduction

Over 60% of pests that affect agriculture and forests are lepidopterans. The silkworm *Bombyx mori* is considered an ideal lepidopteran model insect for scientific research^1^. Moreover, *B. mori* is the main target insect of human domestication and feeding selection in China, India, Uzbekistan, Thailand, and Brazil, and the sericulture industry uses silkworms for the efficient production of silk proteins, which are an important economic resource for more than 30 million households^2^. However, silkworms have not been reared on artificial diets during all-weather factory farming, as are livestock and poultry, as they are still reared widely on fresh mulberry (*Morus*) leaves. This practice has hindered the improvement of sericulture resources in different areas. Industrial pollution and a shortage of labor and mulberry trees have emerged in the traditional sericulture regions, and, especially, the limited harvest season of fresh mulberry leaves has become a bottleneck that hinders the development of modern silkworm breeding farms, and it continues to weaken the viability of sericulture^3–5^. Researchers in Japan and China have been committed to the development of artificial diet-based, silkworm-rearing technologies^3, 5^. Large-scale, artificial diet silkworm rearing was once applied in Japan from the 1980s to the 1990s^6^, but in China, which is the world’s largest sericulture country, artificial diet silkworm rearing has not yet been implemented.

Silkworms are oligophagous insects, and mulberry leaves are their best food source. All nutrients and water silkworms need come from mulberry leaves, which is a result of the long-term co-evolution and natural selection between silkworms and mulberry trees^7, 8^. Nutrients in mulberry leaves that can be used by silkworms are mainly proteins, carbohydrates, lipids, inorganic matter, moisture, and vitamins^9–13^. Although artificial diets for silkworms have mimicked the composition of mulberry leaves, rearing silkworms on artificial diets comprising a large amount of mulberry leaf powder has achieved comparable results to rearing them on mulberry leaves. Most silkworm varieties are not good at ingesting an artificial diet, and they grow and develop poorly as a result; some silkworm varieties do not even ingest an artificial diet^14, 15^. A breakthrough approach to address this issue is the long-term focus of sericulture researchers on breeding silkworm varieties that readily intake artificial diets^16, 17^. However, for almost all silkworm varieties bred so far, only the intake of artificial diets by larvae has been improved, while the metabolic utilization of artificial diets is still not as good as that of mulberry leaves. Issues such as weak silkworm larvae, low silk protein synthesis efficiency, and low silk yields in silkworms reared on artificial diets have not been resolved^6, 18^. Therefore, further improving the artificial diet formula and enhancing its metabolic utilization by silkworms are key issues that must be addressed.

Studies of silkworm artificial diets and nutritional metabolism have focused mainly around the nutritional requirements of silkworms, the nutrient ratio in artificial diets, and screening molding agents and preservatives^11, 13, 19–24^. Meanwhile, a low-cost silkworm artificial diet using cheap livestock feed-based raw materials has also been developed^25–27^. Studies of the conversion and metabolism of nutrients in silkworms, the effect of essential nutrient deficiencies on the blood chemical composition of silkworms, the amount of uric acid excretion, and feed efficiency have also been reported^28–31^. However, little is known about the metabolic differences between silkworms reared on artificial diets and those reared on mulberry leaves, and there is a lack of systematic and comprehensive studies that address this issue. Some urgent questions that need to be answered are: 1) what is the overall difference in nutrient absorption and utilization, as well as nutritional metabolism, between silkworms reared on artificial diets and those reared on mulberry leaves? 2) What is a reasonable explanation, at the metabolic level, why silkworms reared on artificial diets develop poorly and have low silk protein synthesis efficiencies? To answer these questions, here used disruptive selection to produce two genetically stable silkworm strains, one with a high artificial diet intake and another with a low artificial diet intake, from a domesticated silkworm production variety, and we analyzed the metabolomic differences in the hemolymph between silkworm larvae reared on mulberry leaves and those reared on an artificial diet.

## Results

### Impact on an artificial diet on silkworm development and vitality

**Figure 1:**
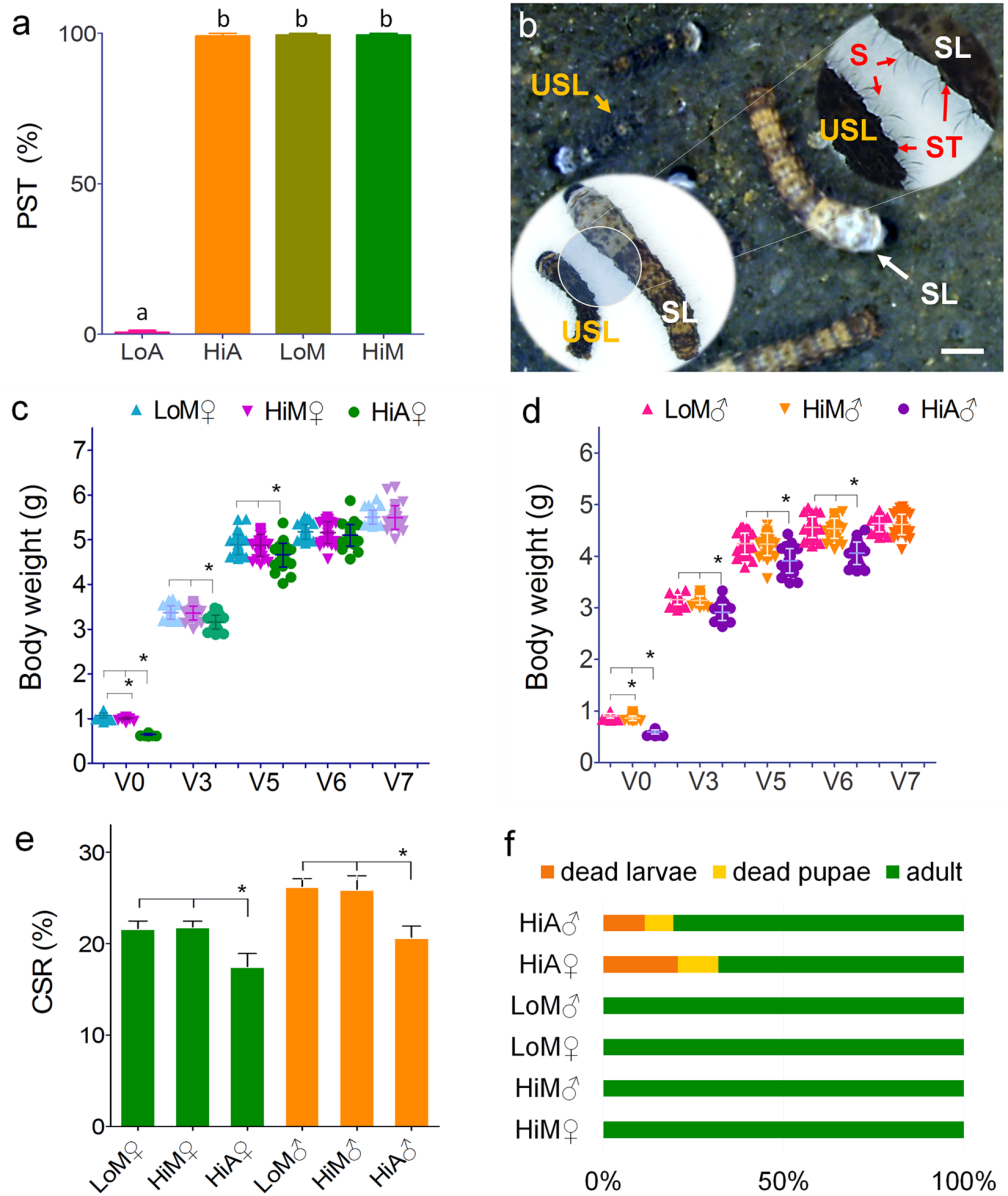
Comparison of the impact of the differences between fresh mulberry leaves and the artificial diet on silkworm development and vitality. (**a**) The percentage of setae dispersion (PST) after feeding for 48 h for newly hatched larvae. (**b**) The setae of scattered larvae (SL) and the setae of unscattered larvae (USL). The bodies of newly-hatched silkworm larvae are black. The larval body is light in color after silkworms eat mulberry leaves or the artificial diet for more than 48 h. Meanwhile, the bulging setae tubercle on the cuticle will flatten. If the larvae cannot eat normally, their growth will be retarded and the bulged setae tubercle on the cuticle will not flatten. The bar = 200 µm. Change of body weight of (**c**) female and (**d**) male fifth-instar larvae (*n* = 30). V0–V7, day 1 to day 7 of the fifth instar. (**e**) The cocoon shell rate (CSR) (*n* = 30). (**f**) The survival rate of the fifth-instar larvae to emergence (*n* = 90). Hi and Lo indicate strains that exhibited high or low ingestion of the artificial diet, respectively. Meanwhile, M and A indicate the larvae reared on fresh mulberry leaves or artificial diet, respectively. ♂, male; ♀, female. Samples marked with the same letter did not differ significantly from each other, *P* > 0.01 (*n* = 100 newly hatched larvae, replicated six times) in Fig. 1a. An *indicates that the difference between the two groups reached the significance level of *P* < 0.01 (*n* = 30) in Fig. 1c,d, and e.

To objectively compare the effect of mulberry leaves and an artificial diet on the growth and vitality of silkworms, after 7 years and 14 generations of continuous selection of the ingestive behavior of the Jingsong B silkworm variety to an artificial diet, we obtained two strains, one of which exhibited high food intake (Hi) and another that exhibited low food intake (Lo). We judged their feeding performance by the percentage of setae dispersion (PST) after feeding for 48 h. The results showed that the PST of the Hi strain reared on the artificial diet (HiA) was close to 100% (Fig. 1a), while that of the Lo strain reared on the artificial diet (LoA) was almost 0%. The Hi and Lo silkworms were further reared on fresh mulberry leaves to generate HiM and LoM silkworms, and their PSTs were almost 100% and were statistically significant different (Fig. 1b).

The growth and development of silkworm larvae were further investigated. The weight gain of fifth instar larvae was used as an index. Whether examining females (Fig. 1c) or males (Fig. 1d), there was no statistically significant difference between the HiM and LoM strains reared on mulberry leaves, but their weights were significantly greater than those reared on the artificial diet. At the same time, the larval stage of silkworms reared on the artificial diet was 12–13% longer than that of those reared on mulberry leaves, while the fifth instar was shortened by 11–14%. The protein synthesis efficiency is represented by the cocoon shell rate (CSR) (Fig. 1e), and the results showed that the CSR of the HiA strain was significantly lower than that of the HiM or LoM strains, although there was no significant difference in the CSR between the HiM and LoM strains. The viabilities of HiA larvae and pupae were significantly lower than those of the HiM and LoM strains (Fig. 1f).

The aforementioned results indicated that the selection of feed intake habits in silkworms did not change the feed intake performance of the experimental varieties on mulberry leaves. The growth, development, viability, and efficiency of silk protein synthesis of the larvae reared on mulberry leaves did not change. However, compared with the silkworms reared on mulberry leaves, these properties of silkworms reared on the artificial diet were significantly worse, suggesting that changes in nutrient absorption and utilization, metabolic conversion, and physical resistance may have occurred in these silkworms.

### Metabolomics changes between silkworms reared on fresh mulberry leaves and the artificial diet

**Figure 2:**
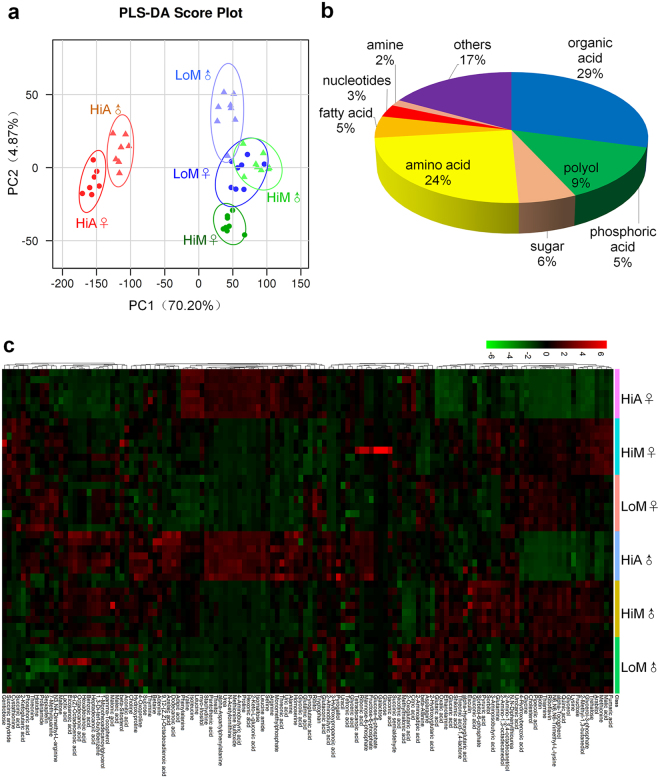
Metabolomics of silkworm larvae reared on fresh mulberry leaves or the artificial diet. (**a**) Partial least squares-discriminant analysis (PLS-DA). (**b**) Classification of metabolites and (**c**) the heat map. Data from the PLS-DA were analyzed using SIMCA-P 13.0 software (Umetrics AB, Umeå, Sweden). The *x*-axis shows the names of the metabolites, and the *y*-axis shows the names of the samples (strain-food-sex). Hi and Lo indicate silkworm strains that exhibited high or low ingestion of the artificial diet, respectively. M, fresh mulberry leaves, *n* = 8 replicates; A, artificial diet, *n* = 7 replicates; ♂, male; ♀, female. Each sample comprised five larvae, which provided an equal volume of hemolymph.

To understand the overall difference in nutritional metabolism between silkworms reared on mulberry leaves and those reared on the artificial diet, the metabolomics of the hemolymph was determined by gas chromatography–mass spectrometry (GC-MS) and liquid chromatography–MS (LC-MS) using 72-h-old fifth instar larvae whose feed intake and nutritional metabolism were the most active. The ion current chromatograms are presented in Figs S1 and S2. The software XCMS (www.bioconductor.org/)^32^ was used to pretreat and analyze the metabolomic data. The partial least squares-discriminant analysis (PLS-DA) score plot (Fig. 2a) showed that there were significant differences in the metabolic characteristics between the HiA and HiM or LoM strains. A cross-validation method was used to test the model quality, and the resulting R2Y and Q2 values were 0.964 and 0.914, respectively, indicating that the model was reliable, which also suggests that the metabolites in the hemolymph of the silkworms reared on the artificial diet differed significantly different from those reared on mulberry leaves.

Two hundred and twenty-eight metabolite peaks detected by GC-MS were further annotated using references in existing databases as well as standards, and 92 substances were annotated successfully. Meanwhile, 46 metabolites were identified using LC-MS; thus, 130 substances were annotated or identified (Table S1). Specific classifications presented in Fig. 2b show that the most abundant differential metabolites were organic acids, followed by amino acids, which accounted for 53% of the identified metabolites, indicating that organic acids and amino acids were metabolized efficiently in the blood of the silkworm larvae.

Figure 2c shows that the most abundant metabolites in the hemolymph of larvae reared on mulberry leaves were quinic acid, followed by citric acid, histidine, methylmalonic acid, N6, N6, N6-trimethyl-L-lysine, serine, glycine, pyroglutamic acid, choline, and alanine. Almost all these compounds are organic acids, amino acids and their intermediates. Meanwhile, the most abundant metabolites in the hemolymph of larvae reared on the artificial diet were citric acid, followed by methylmalonic acid, pyroglutamic acid, serine, histidine, valine, glutaconic acid, choline, leucine, alanine, and quinic acid. These metabolites differed from the former metabolites. Obviously, the metabolism of amino acids and organic acids in the silkworms reared on the artificial diet changed greatly compared with that of the silkworms reared on mulberry leaves, suggesting that the synthesis of silk proteins was also closely related to the diet and food intake of silkworms.

### Analysis of the main differential metabolites

Further analysis of the results shown in Fig. 2c revealed that the difference in the metabolites in the hemolymph of silkworms was not only affected by the nutrient source, either mulberry leaves or the artificial diet, but it was also related to the silkworm strain and gender. Therefore, we first screened 111 differential metabolites between silkworms reared on the artificial diet and those reared on mulberry leaves. Compared with silkworms reared on the artificial diet, the most abundant metabolites in the hemolymph of the silkworms reared on mulberry leaves were thiamine, biotin, pipecolic acid, alpha-tocopherol, quinic acid, cholesterol, and riboflavin. Meanwhile, the most abundant metabolites in the hemolymph of the silkworms reared on the artificial diet were methionine sulfoxide, alpha-aspartylphenylalanine, 2-keto-L-gluconic acid, isomaltose, *N*-acetylornithine, uric acid, and urea. These results suggest that the metabolism of vitamins, nitrogen, and glucose differed significantly in the hemolymph of silkworms that were fed different diets.

**Figure 3:**
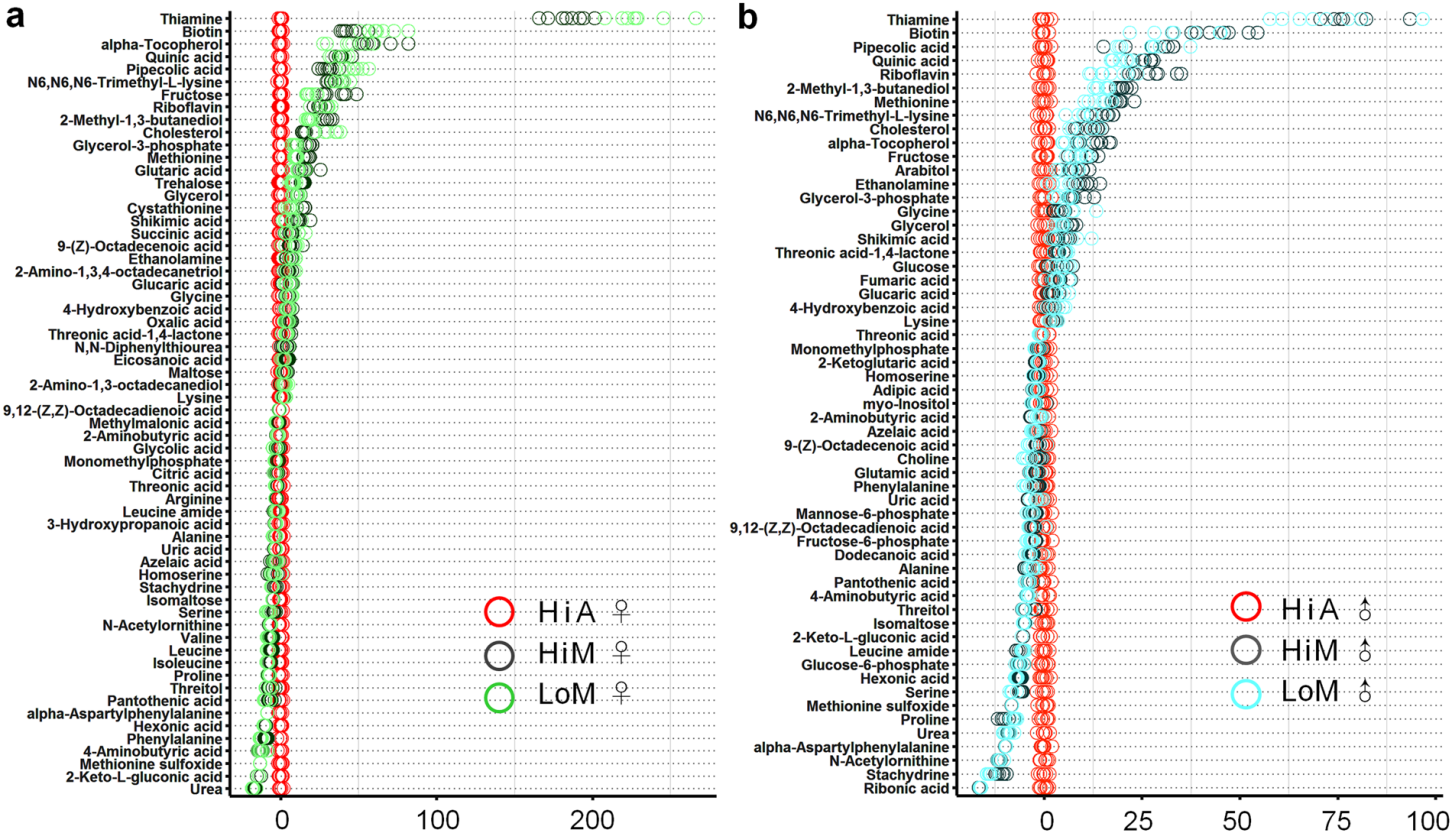
*z*-score plot of the analysis of differential metabolites in the hemolymph between silkworms reared on mulberry leaves and the artificial diet. (**a**) Females and (**b**) males. The *x*-axis shows the *z*-score, and the *y*-axis shows the names of the metabolites. We further screened for differential metabolites, using a partial least squares-discriminant analysis, the first principal component, a variable importance in projection value ≥1, and a *t*-test (*P* ≤ 0.05) as thresholds for the gas chromatography/liquid chromatography-mass spectrometry results. Hi and Lo indicate silkworm strains that exhibited high or low ingestion of the artificial diet, respectively. M, fresh mulberry leaves, *n* = 8 replicates; A, artificial diet, *n* = 7 replicates; ♂, male; ♀, female.

A *z*-score plot analysis of the differential metabolites, shown in Fig. 3, revealed that the metabolites of the two silkworm strains (HiM and LoM) reared on mulberry leaves were similar, and they were stable and consistent within groups, compared with those of silkworms reared on the artificial diet (HiA). Sixty-two and 56 metabolites that exhibited significantly different abundances between the silkworms reared on mulberry leaves and those reared on the artificial diet were identified in female and male silkworms, respectively, and their detailed parameters are listed in Tables S2 and S3, respectively. Using female silkworms, the changes in the abundance of most of the differential metabolites between the HiM and HiA strains were almost identical to those identified between the LoM and HiA strains (Fig. 3a). In the hemolymph of female silkworm reared on mulberry leaves, only the abundances of seven amino acids and organic acids, which were beta-alanine, 3-aminoisobutyric acid, glutamine, pyruvic acid, threonine, benzoic acid, and tryptophan, differed between the HiM and LoM strain (Fig. 3a and Table S4). These results indicate that the differences in metabolites between the female silkworms reared on the artificial diet and those reared mulberry leaves were less affected by the silkworm strains. A similar analysis was performed on male silkworms, and the results also showed that the effect of the silkworm strain on differential metabolites was small (Fig. 3b and Table S5).

Then, the differential metabolites in male and female silkworms that were reared on mulberry leaves or the artificial diet were compared. The results showed that only a few differential metabolites appeared only in one gender, while abundant differences existed in most metabolites between the genders (Fig. 3a and b). An analysis of the more comparable results in Figure S3 showed that the contents of 31 metabolites in female Hi silkworms reared on mulberry leaves were significantly higher than those of silkworms reared on the artificial diet, while the contents of another 31 metabolites were lower (Figure S3a). In the case of male silkworms, the contents of 34 metabolites in the hemolymph of HiM silkworms were significantly higher than those of HiA silkworms, and the contents of another 23 metabolites were lower (Figure S3b). In addition to the four differential metabolites, beta-alanine, 3-aminoisobutyric acid, glutamine, and pyruvic acid, which were affected by the strain of the silkworms in male and female silkworms, 10 other metabolites, including urocanic acid, 2-hydroxyprutaric acid, 3-hydroxypropanoic acid, methylmalonic acid, N, N-diphenylthiourea, sorbitol-6-phosphate, 2-oxoglutaric acid, citric acid, asparagine, and 2-amino-1,3-octadecanediol, in the hemolymph of male silkworms were also affected by the silkworm strain (Fig. 3). These results indicate that gender had a great impact on the metabolites in the hemolymph, and that there was an interactive effect between gender and strain.

**Figure 4:**
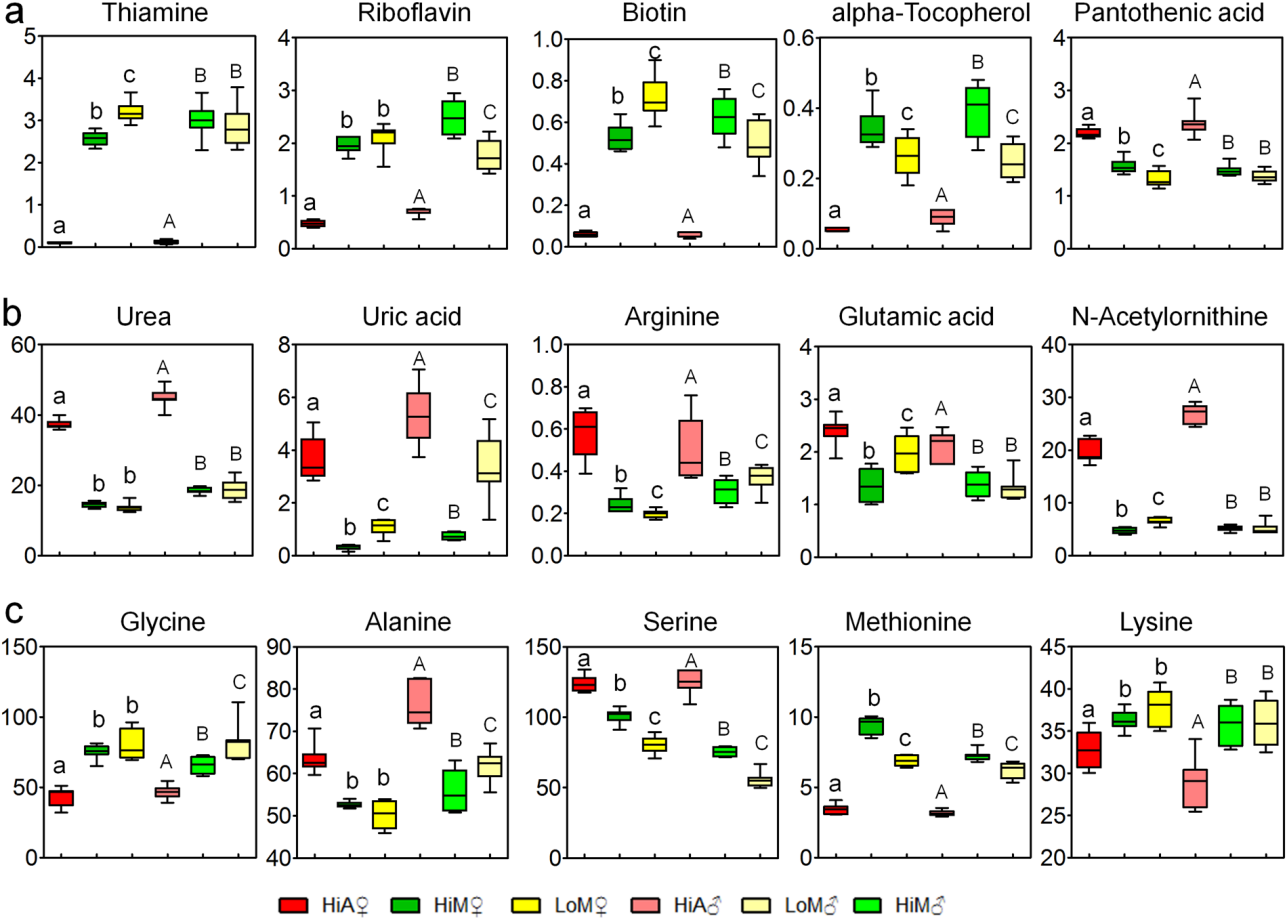
Abundance plot of key metabolites in the hemolymph of silkworms reared on the artificial diet or mulberry leaves. (**a**) Main vitamins, (**b**) main metabolites in the urea cycle, and (**c**) major constituent amino acids in silk protein and some related amino acids. Data were derived from metabolomic assay data, and samples marked with the same letter did not differ significantly from each other (*P* > 0.05). Lowercase (**a**,**b** and **c**) and capital letters (A, B and C) indicate a significant difference between the female silkworm samples and male samples, respectively. Hi and Lo indicate silkworm strains that exhibited high or low ingestion of the artificial diet, respectively. M, fresh mulberry leaves, *n* = 8 replicates; A, artificial diet, *n* = 7 replicates; ♂, male; ♀, female.

An analysis of the main differential metabolites showed that the differences in abundance between the Hi and Lo strains were consistent with those between the male and female genders, while the significant differences in metabolite abundances between silkworms reared on the artificial diet and those reared on mulberry leaves were mainly vitamins, amino acids, and organic acids (Fig. 4). The abundances of vitamins, including thiamine, riboflavin, biotin, and alpha-tocopherol, which play vital roles in the growth and development of larvae, were significantly lower (96.0% in females and 96.1% in males; 75.8% in females and 78.8% in males; 88.7% in females and 91.1% in males, and 84.4% in females and 78.0% in males, respectively), in the hemolymph of the silkworms reared on the artificial diet, compared with those of the silkworms reared on mulberry leaves. Meanwhile, the abundance of pantothenic acid (PA) was 40.4% higher in females and 60.8% higher in males (Fig. 4a).

Another noteworthy feature of the metabolic changes was that the level of uric acid, which is the final product of nitrogen metabolism, was 11.56-fold higher in females (7.1-fold higher in males) in the HiM strain, while the level of urea was 2.52-fold higher in females and 2.44-fold higher in males in the HiM strain (Table S2 and S3). The contents of two key substances, glutamic acid and arginine, in the metabolic pathways of uric acid and urea formation from amino acids were also significantly higher in the hemolymph of the silkworms reared on the artificial diet (Fig. 4b). These results indicate that the levels of uric acid and urea synthesis were significantly enhanced in the hemolymph of the silkworms reared on the artificial diet, suggesting that the utilization of proteins and amino acids in the silkworms reared on the artificial diet was reduced.

Next, we analyzed the changes in amino acid metabolism in the hemolymph of silkworms reared on mulberry leaves and the artificial diet (Fig. 3). Excluding the effect of genetic factors of strain and gender, many amino acid contents in the hemolymph of the silkworms reared on the artificial diet (HiA) differed significantly from those of the silkworms of the same strain and same gender that were reared on mulberry leaves (HiM). The contents of amino acids or their metabolites, including 3-aminoisobutyric acid, N6,N6, N6-trimethyl-L-lysine, methionine, glutamine, glycine, and lysine, were 81.6% lower in females and 89.0% lower in males, 69.8% lower in females and 64.3% lower in males, 63.2% lower in females and 56.3% lower in males, 71.5% lower in females and 77.7% lower in males, 43.2% lower in females and 29.1% lower in males, and 9.9% lower in females and 19.4% lower in males, respectively.

Among the 31 differential metabolites with increased contents in the hemolymph of the female silkworms reared on the artificial diet, 19 were amino acids and their metabolic intermediates, while among the 35 differential metabolites with increased contents in the hemolymph of male silkworms, 17 were amino acids and their metabolic intermediates. The top 15 metabolites with significantly higher abundances in the hemolymph of both male and female silkworms were (in terms of fold-differences) methionine sulfoxide (females 38.11, males 43.19), alpha-aspartyl phenylalanine (females 31.71, males 38.82), *N*-acetylornithine (females 4.12, males 5.19), 4-aminobutyric acid (females 2.59, males 2.68), pyroglutamic acid (females 2.19, males 1.87), proline (females 1.90, males 1.71), glutamic acid (females 1.77, males 1.53), beta-alanine (females 1.40, males 1.52), alanine (females 1.21, males 1.37), 2-aminobutyric acid (females 1.41, males 2.28), and homoserine (females 1.69, males 2.09). Furthermore, the metabolites whose contents in the hemolymph of the female silkworms reared on the artificial diet were significantly higher than those of the silkworms reared on mulberry leaves were, in terms of fold-differences, leucine (1.41), isoleucine (1.65), asparagine (1.69), and tryptophan (1.26); however, these metabolites did not exhibit any statistically significant differences between male silkworms reared on the artificial diet and mulberry leaves (Tables S2 and S3). It is worth noting that the contents of glycine, alanine, and serine, the main amino acids in silk protein, differed significantly in the hemolymph of the silkworms reared on the artificial diet. The glycine content was significantly lower than that of the silkworms of the same strain reared on mulberry leaves, while the alanine and serine contents were significantly higher (Fig. 4c). These results indicate that there was a problem regarding the supply and utilization of amino acids, the raw materials used for silk protein synthesis, in the silkworms reared on the artificial diet.

An analysis of the aforementioned results showed that although the feed intake habits of the two silkworm strains reared on the artificial diet were very different, they were bred using the same silkworm variety by selecting for their adaptability to the artificial diet only; the differences in growth, development, and basal metabolism were very small between the two strains. Therefore, in a follow-up analysis, we focused on the metabolic differences between the two genders, and the metabolic differences between silkworms of the same strain reared on the artificial diet or mulberry leaves.

**Figure 5:**
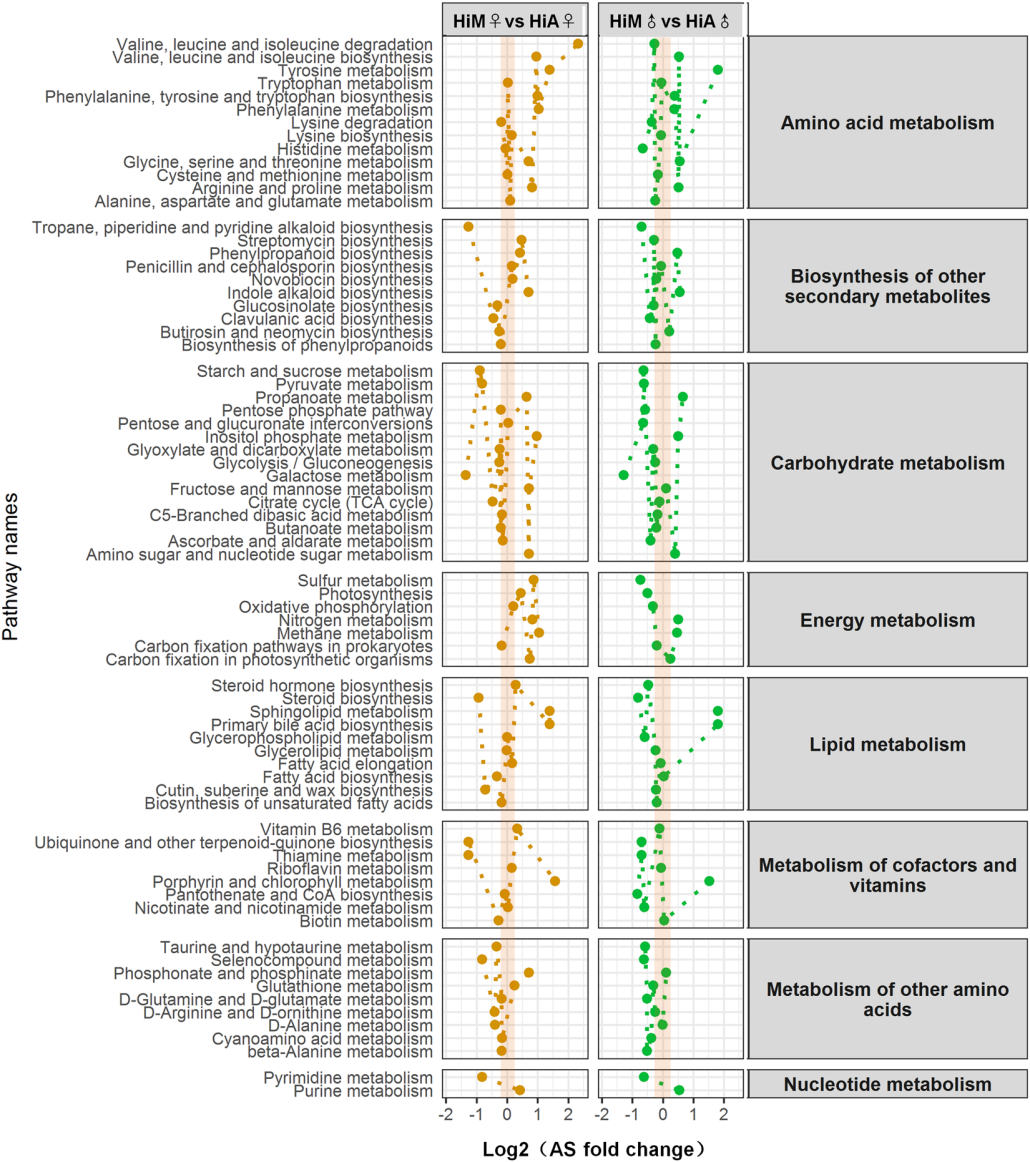
Heat map analysis of differential metabolic pathways in silkworms reared on the artificial diet or mulberry leaves. The Hi silkworm strain was used for the comparison between genders. The analysis was based on a visual analysis of metabolic pathways^62^. A pathway activity profiling (PAPi) algorithm was used to calculate the activity score (AS) for each metabolic pathway, and the calculated PAPi and the relative abundance of each metabolic pathway were based on the Kyoto Encyclopedia of Genes and Genomes (KEGG) database. The metabolic pathway heat map was produced based on the KEGG metabolic process classification, and an analysis of variance was used for the statistical test (*P* < 0.05 indicates significance). Different circles represent the AS values of different metabolic pathways.

To gain a comprehensive understanding of hemolymph function, we performed a Kyoto Encyclopedia of Genes and Genomes (KEGG) pathway analysis of the hemolymph proteins using the Molecule Annotation System (MAS 3.0, http://bioinfo.capitalbio.com/mas3/). The results showed that amino acid metabolism, biosynthesis of other secondary metabolites, carbohydrate metabolism, energy metabolism, lipid metabolism, and metabolism of cofactors and vitamins in the hemolymph of the female silkworms reared on the artificial diet differed significantly compared with the female silkworms of the same strain reared on mulberry leaves, indicating that there were differences in multiple metabolic pathways between the silkworms reared on the artificial diet and those reared o mulberry leaves (Fig. 5). In addition to vitamins whose differences were significant, other metabolites with significant metabolic differences included aspartic acid, alanine, and glutamic acid among amino acid metabolism, and the glycolysis and the tricarboxylic acid cycle (TCA) cycle of carbohydrate metabolism, while in nucleotide metabolism, only purine metabolism differed significantly. Lipid metabolism was less affected, as only the synthesis pathways of cholesterol and steroid hormones were affected, while the contents of cholesterol and glycerol were lower. At the same time, the contents of organic acids, such as pipecolic acid, quininic acid, shikimic acid, pyruvic acid, and fumaric acid, which play extremely important roles in metabolism, were also very low in the hemolymph of the HiA silkworms.

### Analysis of differential metabolites between males and females

**Figure 6:**
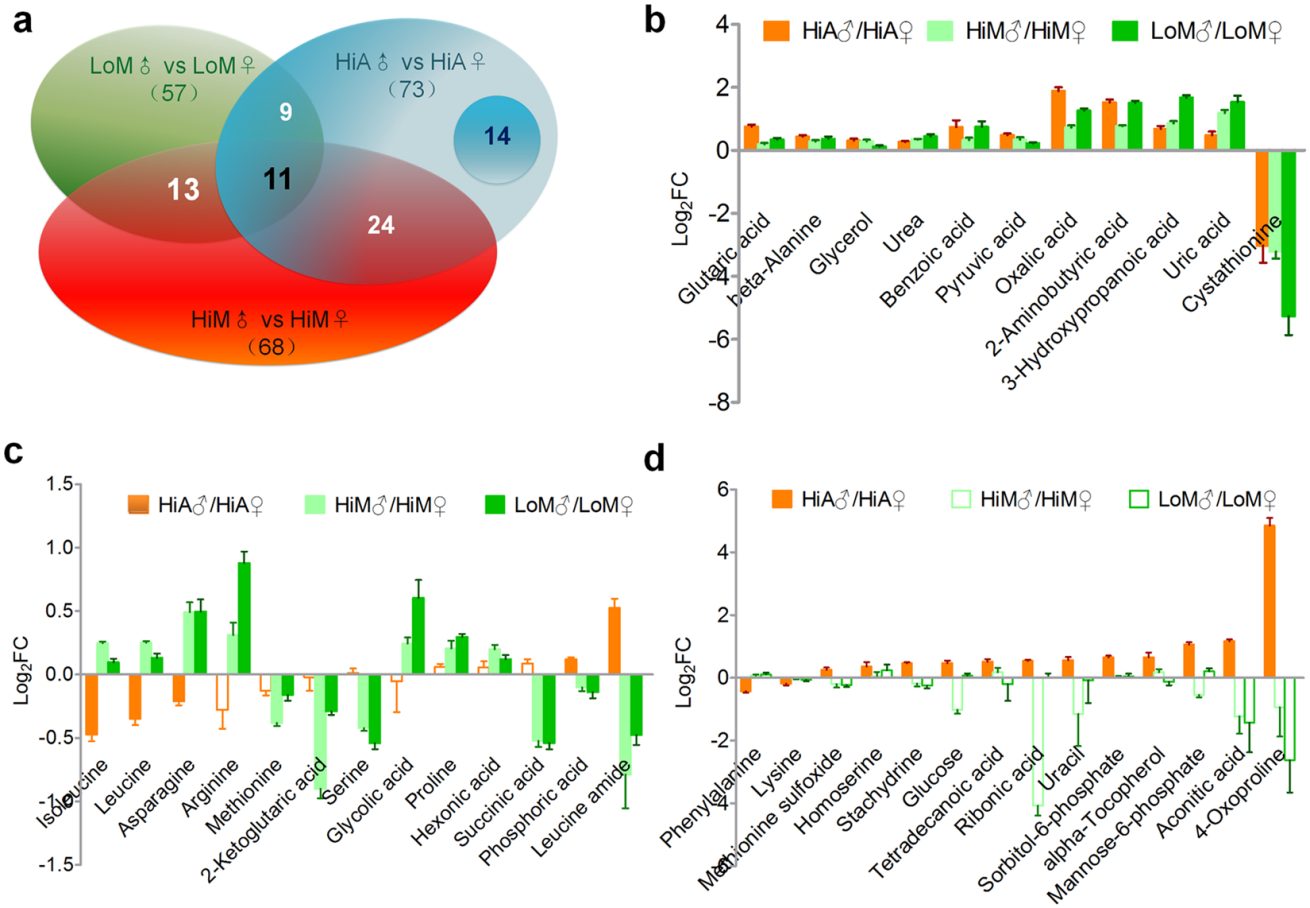
Comparison of differential metabolites between genders. (**a**) The number of differential metabolites between genders in different silkworm strains that were fed different diets. (**b**) Metabolites with stable differences (*P* < 0.05) between genders in both silkworm strains reared on the two diets. (**c**) Metabolites exhibiting significant differences in abundance between the hemolymph of silkworm genders reared on mulberry leaves (*P* < 0.05), but without significant differences between genders in the silkworms reared on the artificial diet, and metabolites exhibiting significant differences in abundance between the genders in the silkworms reared on the artificial diet, but with opposite patterns compared with those reared on mulberry leaves. (**d**) Metabolites with significant differences in abundance in the hemolymph between genders of the silkworms reared on the artificial diet (*P* < 0.05), but without significant differences between genders in the silkworms reared on mulberry leaves (*P* ≥ 0.05). FC indicates the fold-change of the relative abundance (male/female). No fill color indicates that there was no significant difference in metabolite abundance between males and females.

To analyze the metabolic differences between silkworms of different genders, we analyzed the differential metabolites between genders in silkworms reared on mulberry leaves or the artificial diet based on GC/LC-MS data. The number of differential metabolites between male and female HiA silkworms reared on the artificial diet was 73, while the numbers of differential metabolites between males and females in the hemolymph of silkworms of the HiM and LoM strains reared on mulberry leaves were 68 and 57, respectively (Fig. 6a).

Eleven metabolites with stable differences (*P* < 0.05) between genders, regardless of strain or diet, were further screened (Fig. 6b). Among them, the abundance of cystathionine in the hemolymph of female silkworms was significantly higher than that in male silkworms (13.97-, 14.79-, and 5.11-fold higher in the LoM, HiM, and HiA strains, respectively, which is consistent with the fact that cystathionine is involved in the formation of silkworm eggs^33^. While the abundances of 10 other metabolites, which were mainly organic acids such as uric acid and oxalic acid, in the hemolymph of male silkworms were significantly higher than those in female silkworms.

Figure 6c shows that there were significant differences in metabolite abundance in the hemolymph between males and females (*P* < 0.05) reared on mulberry leaves (HiM and LoM) or the artificial diet (HiA). Five metabolites, including three amino acids (leucine, isoleucine, and asparagine), exhibited higher contents in the male silkworms reared on mulberry leaves and the female silkworms reared on the artificial diet, while the leucine amide and phosphoric acid contents exhibited the opposite pattern. The abundance of eight other metabolites, including four amino acids (proline, arginine, serine, and methionine) and four important organic acids related to energy metabolism (hexonic acid, glycolic acid, succinic acid, and 2-ketoglutaric acid) differed significantly different between the male and female silkworms reared on mulberry leaves (*P* < 0.05), but not between the male and female silkworms reared on the artificial diet (*P* ≥ 0.05).

We also screened 14 metabolites that had significant abundance differences in the hemolymph between the male and female silkworms reared on the artificial diet (HiA) (*P* < 0.05), but no significant differences in the hemolymph between the male and female silkworms reared on mulberry leaves (HiM and LoM) (*P* ≥ 0.05) (Fig. 6d). They included five amino acids and their related metabolites (lysine, phenylalanine, methionine sulfoxide, homoserine, and 4-oxoproline), five carbohydrates and organic acids related to energy metabolism (glucose, tetradecanoic acid, sorbitol-6-phosphate, mannose-6-phosphate, and aconitic acid), two metabolites related to nucleic acid metabolism (ribonic acid and uracil), and a vitamin and an alkaloid (alpha-tocopherol and stachydrine, respectively).

## Discussion

GC-MS is suitable for the analysis of small-molecule, thermostable, volatile, gasifiable compounds, while LC-MS can analyze compounds with higher polarity, higher relative molecular mass, and poor thermal stability. Numerous studies have shown how these techniques may be used to complement each other^34–36^. Zhou *et al*.^9^ used nuclear magnetic resonance spectroscopy and GC-flame ionization detector/MS to investigate the hemolymphatic metabolomics of the silkworm P50 strain during different developmental stages^9^. Their results showed that the major metabolites in larval hemolymph are highly similar to those that we identified from silkworms reared on fresh mulberry leaves, and they suggest that the metabonome in hemolymph exhibits outstanding stability in different strains reared on mulberry leaves. This may be the result of the evolutionary adaptation of domesticated silkworms. GC-MS was applied to identify key metabolic changes in three kinds of silkworm female larvae in which silk gland development exhibits obvious differences^37^. Their results showed that the levels of glycine and uric acid were significantly upregulated, while those of carbohydrates and free fatty acids were significantly downregulated in silkworm larvae that have vestigial silk glands or silkworms that have had their silk glands removal. These results are similar to ours, which showed that the silk protein synthesis efficiency decreased in the larvae reared on the artificial diet, and they suggest that the increase of glycine and uric acid metabolism may be related to silk protein synthesis efficiency. In the present study, we used GC/LC-MS to investigate metabolomics differences between silkworms reared on fresh mulberry leaves or artificial diets.

### Changes of vitamin metabolism in silkworms reared on the artificial diet

**Figure 7:**
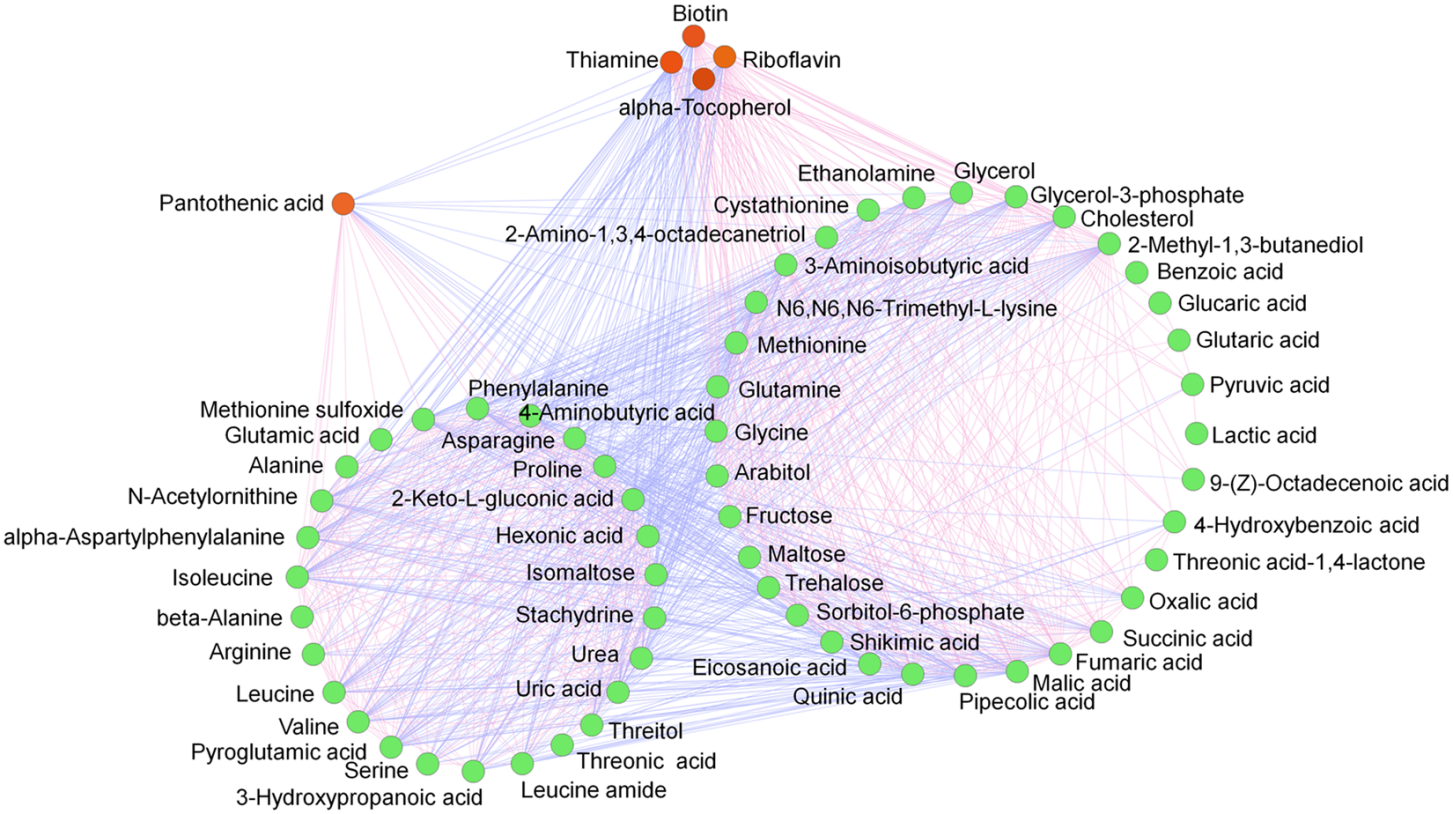
Correlation network of differential metabolites. Pearson’s correlation coefficient was used to analyze correlations among metabolites, and the calculation was performed using the cor() function in the R language package (www.r-project.org). The Pearson correlation coefficient threshold was set to 0.9. Red lines represent positive correlations between substances, and blue lines represent negative correlations between substances.

Silkworms reared on the artificial diet were lighter, less vital, and produced less silk than those reared on mulberry leaves. These results have been reported by many researchers studying silkworm artificial diets, although the reason remains unclear^6, 18, 38, 39^. The results of the metabolomic analysis in this study showed that the contents of vitamins, including thiamine, biotin, alpha-tocopherol, and riboflavin, in the hemolymph of silkworms reared on the artificial diet were significantly lower than those of silkworms reared on mulberry leaves, and these differences were still significant after excluding the effect of genetic factors of silkworm strain and gender. A correlation network analysis of differential metabolites of these vitamins was conducted using Pearson’s correlation coefficient, and 59 differential metabolites (33 + 26) constituted many edges, forming a complex topological network (Fig. 7). According to the “cluster factor” concept^40^, the more linkages a metabolite has, the greater its cluster factor. Therefore, we can assume that these vitamins play important roles as information linkages in the topological structure, suggesting that they may play important roles in metabolic regulation. Previous reports showed that thiamine is classified as an essential, water-soluble vitamin that requires continuous dietary intake to support carbohydrate metabolism. Thiamine is critical for the activity of four key enzymes in cellular metabolism: pyruvate dehydrogenase and alpha-ketoglutarate dehydrogenase in the TCA acid cycle, transketolase within the pentose phosphate pathway, and a branched-chain alpha-keto acid dehydrogenase complex that is involved in amino acid catabolism^41^. Biotin is a water-soluble vitamin that is a cofactor for carboxylase in fatty acid synthesis, amino acid metabolism, and gluconeogenesis^42, 43^. Alpha-tocopherol has a great ability to clear reactive oxygen species^44^, and a riboflavin deficiency can cause a downregulation of immune function^45^, leading to increased susceptibility to infection^46^.

We found that only the content of PA was significantly higher in silkworms reared on the artificial diet, while the contents of various vitamins were lower (Fig. 4). PA is a component of coenzyme A, and the coenzyme form of PA is involved in acyl group transfer reactions, the TCA cycle, and choline actelylation^47^. Although the optimal concentration of PA can promote animal growth, it has been reported that a deficiency or excess of PA will reduce an animal’s antioxidant capacity^48^.

Sericulture researchers have focused their attention on the effects of vitamins on the growth and development of silkworms for a long time^11, 13^. Currently, although multivitamins are generally added to commercially available silkworm feed formulae, high-temperature sterilization treatment of the feed before feeding silkworms may lead to the loss of some vitamins. Therefore, exploring a more effective method for vitamin addition in silkworm feed is an issue that should receive greater attention in sericulture in the future.

### Other features of metabolic changes in silkworms reared on the artificial diet

**Figure 8:**
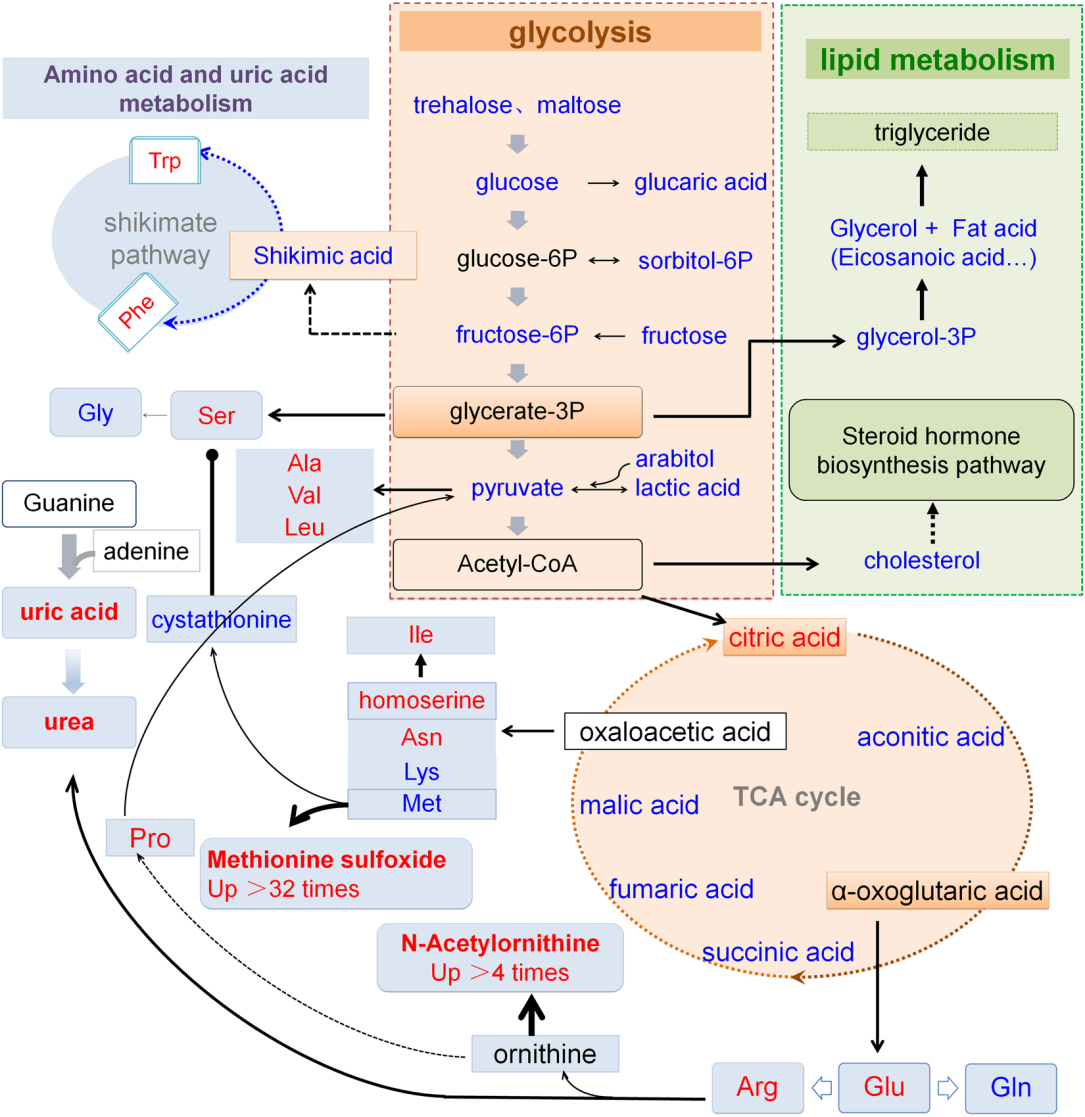
Summary of the primarily affected metabolic pathways in the hemolymph of silkworms reared on the artificial diet. The pathways were simplified and organized based on Kyoto Encyclopedia of Genes and Genomes pathways (http://www.kegg.jp/). The data were collected from the metabolomic data of female larvae of the high feed intake strain reared on the artificial diet (HiA females). The font color represents the level of change in the silkworms reared on the artificial diet compared with that of the silkworms of the same strain reared on fresh mulberry leaves (HiM females); red indicates a higher level; blue indicates a lower level; and black indicates no significant change. Black font with a black frame indicates an unidentified metabolite.

Based on the KEGG (http://www.kegg.jp/) metabolic pathways, we summarized the metabolic pathways of differential metabolites involved in glycolysis, lipid metabolism, amino acid metabolism, and uric acid metabolism in the hemolymph of Hi female silkworms reared on the artificial diet or mulberry leaves. The results in male silkworms (Figure S4) were highly similar to those in the females. Figure 8 shows that among the metabolic pathways in female silkworms, there were some differential metabolic pathways in the hemolymph of silkworms reared on mulberry leaves or the artificial diet that are worthy of attention.

### Amino acid and uric acid metabolism

A conundrum in sericulture is that even if a high-protein diet is given to silkworms, their silk protein synthesis efficiency does not improve. The results in Fig. 3 show that the contents of intermediates of amino acid metabolism, including alanine, serine, and beta-alanine, in the hemolymph of silkworms reared on the artificial diet were significantly higher than those of silkworms reared on mulberry leaves, among which, the methionine sulfoxide content in the hemolymph of silkworms reared on the artificial diet was 31.7-fold higher in females and 43.2-fold higher in males, while the alpha-aspartyl phenylalanine content was 38.1-fold higher in females and 38.8-fold higher in males, while the contents of six other amino acids and their intermediates, including glycine, were significantly lower than those of silkworms reared on mulberry leaves. It is worth noting that the contents of essential amino acids that are required for the development of silkworms, including lysine and methionine, were 63.2% lower in females (56.3% lower in males) and 9.9% lower in females (19.4% lower in males), respectively.

The shikimic acid metabolic pathway is only found in higher plants, fungi, and bacteria^49^. Animals cannot synthesize three aromatic essential amino acids, phenylalanine, tyrosine, and tryptophan, through the shikimic acid metabolic pathway, so these three amino acids must be supplied in their diet^50^. Quinic acid is present in the artificial silkworm diet, and the precursor for tyrosine and phenylalanine is present in mulberry leaves^9^. In this study, abundant amounts of shikimic acid and quinic acid were detected in the hemolymph of silkworms reared on mulberry leaves. However, as the content of shikimic acid was significantly lower in the hemolymph of silkworms reared on the artificial diet, the contents of phenylalanine and tryptophan, which are essential amino acids for silkworms that are synthesized via this pathway, were significantly higher than those of silkworms reared on mulberry leaves (Fig. 8), while only the phenylalanine content in male silkworms reared on the artificial diet was significantly higher than that of silkworms reared on mulberry leaves (Table S1).

Studies have reported that a lack of any essential amino acids in the diet will lead to the inhibition of protein synthesis, which results in increased free amino acids in the blood and increased excretion of uric acid and urea^51^. It has also been reported that excessive amino acid levels in the diet lead to decreased silkworm vitality. The results in this study suggest that the slow growth and low viability of silkworms reared on the artificial diet were not only related to the low absorption rate of some essential amino acids^52^, but may also be affected by the high levels of amino acids and their intermediates in the hemolymph.

Silkworms excrete uric acid and excess amino acids, resulting either from food intake or generation by *in vivo* metabolism, mostly through the Malpighian tubules, or they accumulate in the epidermis in the form of ammonium salt, while small amounts of amino acids are converted to uric acid or urea through purine metabolism^53^. Our results showed that the contents of uric acid and urea in the hemolymph of silkworms reared on the artificial diet were significantly higher than those of silkworms reared on mulberry leaves (Figs 4 and 8), which may be related to the deficiency of a high-activity urease, which exists in fresh mulberry leaves, but cannot be synthesized by silkworms^54^. Further analysis of the results in Fig. 8 showed that the contents of 2-oxoglutaric acid, glutamic acid, and arginine, three key substances in the metabolic pathways leading from amino acids to uric acid and urea, were significantly higher in the hemolymph of silkworms reared on the artificial diet. This can be explained because amino acids with high abundances in the hemolymph of the silkworms reared on the artificial diet, such as glutamate and arginine, were utilized rapidly in the uric acid and urea synthetic pathways, rather than normally during the synthesis of proteins that are needed for growth or silk protein production in the silkworms reared on mulberry leaves.

At the same time, the contents of alanine, glycine, asparagine, and serine, which are the most abundant amino acids, in silk protein, differed significantly in the hemolymph of silkworms reared on the artificial diet. These four amino acids accounted for 85.5% of the amino acids in silk fibroin and 71.6% in sericin, and the glycine content in silk fibroin reached 46.5%^55, 56^. These results show that the abundance of glycine in the hemolymph of the silkworms reared on the artificial diet was 43.5% lower in females and 29.0% lower in males compared with those of the silkworms reared on mulberry leaves, while the contents of alanine, asparagine, and serine were significantly higher, suggesting that the low efficiency of silk protein synthesis in the silkworms reared on the artificial diet may be related to the lack of glycine.

It has been reported that glycine plays an important role in regulating silk synthesis in silkworms^37^. In our study, the glycine level was significantly lower in the silkworms reared on the artificial diet compared with those reared on mulberry leaves, which may be related to the low silk protein synthesis efficiency. Although the existing artificial diet formula has undergone many optimizations, its amino acid ratio has not been optimized based on metabonomics. Hence, silkworms reared on the artificial diet may experience amino acid metabolism disorders to a certain degree. This problem cannot be expected to improve the glycine content of the feed solution, but it should be based on metabolomics and the optimization of the amino acid (protein) formula in the silkworm feed.

### Carbohydrate metabolism

The level of carbohydrate metabolism in silkworms reared on the artificial diet was lower than that in those reared on mulberry leaves. The levels of important metabolites of glycolysis, including trehalose, glucose, fructose, gentiobiose, and pyruvic acid, were significantly lower, and the contents of malic acid, succinic acid, and fumaric acid in the TCA cycle were also significantly lower (Fig. 8) in both genders. The TCA cycle is a common pathway for carbohydrate, lipid, and amino acid metabolism; therefore, the decrease of the level of carbohydrate metabolism in the silkworms reared on the artificial diet resulted in an insufficient energy metabolism, and even further led to the accumulation of some amino acids.

Carbohydrate requirements are also related to the contents of proteins and amino acids. However, the data in this study showed that the contents of some carbohydrate metabolites, such as isomaltose, citric acid, and 2-oxoglutaric acid, in the hemolymph of silkworms reared on the artificial diet were significantly higher, suggesting that the metabolic utilization of proteins and amino acids, and even the change of the lipid anabolic level, may be related to the change of carbohydrate metabolism.

Carbohydrates obviously promote food intake in silkworms. There was no significant difference in the content of sucrose, the most efficient sugar that promotes food intake in silkworms, in the hemolymph between silkworms reared on the artificial diet and those reared on mulberry leaves, but the fructose content was significantly lower in the silkworms reared on the artificial diet, which may be related to their decreased feed intake.

### Lipid and cholesterol metabolism

Sterols are essential nutrients for silkworms and other insects. Silkworms must acquire sterols continuously from their food for their growth and development. A sterol deficiency will affect ecdysone synthesis and oviposition, while having sufficient sterols, but lacking fatty acids, also restrains the growth of silkworms^57, 58^. In this study, the difference of fatty acid metabolism in the hemolymph between male and female HiA silkworms was obvious compared with that of HiM silkworms of the same strain and same gender reared on mulberry leaves. The contents of three free fatty acids, heptadecanoic acid, octadecanoic acid, and eicosanoic acid, were significantly lower, while the dodecanoic acid and hexonic acid contents were significantly higher in HiA females. However, in HiA males, no lower fatty acid contents were detected, and the contents of fatty acids, including dodecanoic acid, tetradecanoic acid, hexonic acid, octadecanoic acid, and eicosanoic acid, were significantly higher. It was also found that the cholesterol content in the silkworms reared on the artificial diet was significantly lower in females (62.0%) and males (71.4%) compared with those of the silkworms reared on mulberry leaves. These results indicate that a significant change in lipid metabolic pathways also existed in the silkworms reared on the artificial diet.

### Gender differences in silkworm metabolism

Male *B. mori* larvae are superior to females in terms of integrated economic traits, such as resistance to cytoplasmic polyhedrosis virus, feed efficiency, silk protein synthesis efficiency, and silk quality^59, 60^. In this study, 11 significantly differential metabolites between genders were screened to exclude the effects of genetic factor (strain) and diet (mulberry leaves and artificial diet) differences (Fig. 6). The abundances of 10 of the 11 metabolites, including uric acid, 3-hydroxypropanoic acid, 2-aminobutyric acid, pyruvic acid, oxalic acid, benzoic acid, urea, glycerol, beta-alanine, and glutaric acid, in the hemolymph of male silkworms were significantly higher than those in females. These are characteristic male metabolites in silkworms that have not yet been reported. Most of these metabolites are intermediates and final products (uric acid, urea) of amino acid metabolism, indicating that the accumulation of amino acid metabolites and the production of excreted nitrogen were higher, while amino acid utilization was lower, and the inhibition of protein synthesis was greater in male silkworms than females.

In this study, we further screened five metabolites whose abundances in hemolymph differed between male and female larvae reared on mulberry leaves, while differences in their abundances between genders exhibited an opposite pattern when larvae were reared on the artificial diet. Furthermore, 22 metabolites exhibited significant differences in abundance between genders only in silkworms reared on either mulberry leaves or the artificial diet. These differential metabolites comprised 13 amino acids and their related metabolites, including serine, lysine, proline, and arginine, and 10 important carbohydrates and organic acids, including glucose, succinic acid, ribonic acid, and uracil that are involved ribonucleic acid metabolism, as well as vitamins and alkaloids, including stachydrine and alpha-tocopherol. The presence of these differential metabolites may be associated with the difference in adaptability to the artificial diet between the genders, as well as the gender differences in nutritional metabolism, silk protein synthesis, and physical properties when the silkworms were reared on the artificial diet.

In closing, to compare and analyze the metabolomics of silkworms reared on fresh mulberry leaves or artificial diets, we showed that a serious shortage of some vitamins, as well as downregulations of glycolysis, the TCA cycle, and lipid metabolism occurred in the silkworms reared on artificial diets. Meanwhile, the levels of multiple amino acids and amino acid-related metabolites increased in these larvae. Hence, future studies should focus on the optimization of the constituents of artificial diets based on a comprehensive performance of feedstuff. Our results suggest that focusing on the amino acid balance and exploring a more effective method for vitamin addition in artificial diets could lead to important breakthroughs. Furthermore, genetically modifying target genes that regulate silk synthesis of silkworms is an issue that should receive greater attention in sericulture in the future.

## Materials and Methods

### Preparation of animals

In this study, using a combination of batch selection and individual selection, a continuous, we conducted a multi-generational, disruptive selection of the ingestive behavior of silkworm larvae to an artificial diet. The starting material was a domesticated silkworm variety, *B. mori* Jingsong B (Chinese system). We judged feeding performance by the PST after feeding for 48 h (Table S6). The bodies of newly-hatched silkworm larvae are black. The bodies should be light in color after normal feeding on mulberry leaves or the artificial diet for more than 48 h. Meanwhile, the bulged setae tubercle on the cuticle becomes flattened. If the larvae cannot eat normally, their growth will be retarded, and the bulged setae tubercle on the cuticle will not flatten. After 7 years of continuous selection of 14 generations, we obtained two strains: in one the strains, almost 100% of the larvae ingested the artificial diet, while almost 100% of the larvae in the other strain did not ingest the artificial diet and starved to death during their newly hatched larval stage. They are named the high ingestive habit strain and the low ingestive habit strain, which are abbreviated as Hi and Lo, respectively. The Hi and Lo strains share the same genetic background.

Larvae of both stains were reared on fresh mulberry leaves, while the larvae of the Hi strain were reared on the artificial diet at 25 °C with a 12 h light/12 h dark photoperiod. During the fifth instar, male and female individual body weights (*n* = 30) and the survival rate from the fifth instar larva to emergence (*n* ≥ 90) were recorded every 24 h. The CSR (*n* = 30) was examined at day 9 after spinning. Similar-sized larvae were selected to assess their metabolomics. After cutting the anal horn of the larvae, a hemolymph sample was collected from five male or female larvae, which provided an equal volume of hemolymph, and it was tested eight times. Because the fifth-instar developmental stage is a vital life stage for silk protein synthesis by silkworms, and the hemolymph can reflect the global metabolic status, the hemolymph of silkworm larvae was collected on ice 72 h into the fifth instar, although thiourea was not added. Then, the samples were placed rapidly in liquid nitrogen and stored at −80 °C prior to the metabolomics analysis by GC/LC-MS.

### Preparation of mulberry leaves and diet

The artificial diet composition (w/w) was as follows: 35% mulberry leaf powder, 35% soybean powder, 15% mulberry green twig powder, 9.4% starch, 1.5% vitamin C, 1.5% vitamin B complex, 2% citric acid, 0.4% crotonic acid, and 0.2% choline chloride. The powdered ingredients were mixed with 1.9 times (w/w) as much UP water, and the mixture was placed into 25 × 30 cm preservation bags. The bags were pressed to a thickness of approximately 3 mm, sealed, and then boiled at 100 °C for 50 min. The bags were cooled naturally and preserved at 4 °C. Fresh mulberry leaves were picked from mulberry trees of the Husang strain on the Dushuhu campus of Soochow University.

### GC/LC-MS assay

GC-MS and LC-MS were conducted at BioNovoGene Co., Ltd. (Suzhou, China). One hundred microliters of silkworm blood was collected, and 400 μL of methanol was added, followed by mixing for 1 min. Then, internal standards of 60 μL of 2-chlorophenylalanine (0.2 mg/mL) and 60 μL of heptadecanoic acid (0.2 mg/ml) were added, and the mixture was vortexed for 60 s and centrifuged at 13,000 × *g* for 10 min at 4 °C. The supernatant was transferred to a new centrifuge tube and concentrated in a vacuum centrifugal concentrator. Then, 90 μL of methoxyl solution was added, and the mixture was vortexed for 30 s and then incubated at 37 °C for 2 h. Finally, 60 μL of *N*, *O*-bis (trimethylsilyl) trifluoroacetamide (containing 1% trimethylchlorosilane) was added, and the mixture was incubated at 37 °C for 90 min. The mixture was centrifuged at 13,000 × *g* for 10 min at 4 °C and the supernatant was collected. The resulting samples were subjected to GC-MS (Agilent 7890 A/5975 C) using an HP-5MS capillary column (5% phenylmethylsiloxane: 30 m × 250 μm internal diameter, size 0.25 μm; Agilent, J & W Scientific, Folsom, CA, USA). The parameters for the detection are shown in Figure S1.

One hundred microliters of blood samples was collected, and 200 μL of methanol was added, followed by mixing for 1 min and centrifugation at 13,000 × *g* for 10 min at 4 °C. Then, the supernatant was filtered through a 0.22-μm membrane, and subjected to LC-MS (Waters UPLC, Waters, Milford, MA, USA) using a C18 column (1.7 μm, 2.1 × 100 mm) (Ethylene Bridged Hybrid, Waters). The parameters for the detection are shown in Figure S2.

### Data statistics

The original GC-MS and LC-MS data were converted into a format recognized by the XCMS program (www.bioconductor.org/)^32^, and they were analyzed at BioNovoGene Co., Ltd. In addition to the XCMS default parameters, the following adjustments were made: xcmsSet (fwhm = 3, snthresh = 3, mzdiff = 0.5, step = 0.1, steps = 2, max = 300), retcor (method = “obiwarp”, plottype = c (“Deviation”), bandwidth (bw) is 2, minfrac = 0.3. The final results of the XCMS were derived from EXCLE for our further analysis.

The metabolite annotation of the GC-MS data was performed with an automatic processing and identification system (AMDIS), referenced to the databases of the National Institute of Standards and Technology and the Wiley Registry of Mass Spectral Data (Wiley Online Library). The metabolite annotation of the LC-MS data was performed with the Compound Discoverer 2.0 program and referenced to the mzCloud database (www.mzCloud.org), as well as the Human Metabolome Database (http://www.hmdb.ca website), METLIN (http://metlin.scripps.edu/website), MassBank (http://www.massbank.jp/), and LIPID MAPS (http://www.lipidmaps.org).

Differential metabolites between groups were found using a PLS-DA, which is a multivariate statistical method for discriminant analysis, based on the first principal component a Variable Importance in Projection (VIP) value, VIP > 1, and a Student’s *t*-test (*P* < 0.05). Statistical tests were performed using R 3.0.3 software. PLS-DA was used for the supervised data analysis, The Pheatmap function of the R language package was used for the heat map analysis (www.r-project.org).

Pearson’s correlation coefficient was used for the metabolite correlation analysis. The linear correlation of the abundance of metabolites between silkworms reared on the artificial diet or mulberry leaves was measured using Pearson’s *r*.

The calculation method used the cor() function in the R language package (www.r-project.org), and a false-positive check was performed on *P* using False Discovery Rate (FDR) *P ≤ *0.05. The relationship among metabolites for constructing metabolic pathways was based on the KEGG database^61^ (http://www.genome.jp/kegg/). A univariate analysis of variance was used to determine significance differences in relative contents between different groups.

A Pathway Activity Profiling (PAPi) analysis of the metabolic pathways was performed to identify differential metabolites^62, 63^. The PAPi algorithm was used to calculate the activity score (AS) for each metabolic pathway, and the calculated PAPi and the relative abundance of each metabolic pathway were based on the Kyoto Encyclopedia of Genes and Genomes (KEGG) database (http://www.kegg.jp/). PAPi package is available in: http://www.4shared.com/file/s0uIYWIg/PAPi_10.html. The *P*-value of each metabolic pathway was calculated using a *t*-test or analysis of variance, and metabolic pathways with *P* < 0.05 were retained.

## Electronic supplementary material

Supplementary Information

Table S1
