# Supplementary material for: Metabolomics differences between silkworms (Bombyx mori) reared on fresh mulberry (Morus) leaves or artificial diets
Source: Sci Rep. 2017 Sep 8;7:10972. doi: 10.1038/s41598-017-11592-4 (PMC5591246; doi:10.1038/s41598-017-11592-4)
Supplement: Supplementary file 1 — Supplementary Information [file 41598_2017_11592_MOESM1_ESM.pdf]

Metabolomics differences between silkworms (*Bombyx mori*) reared on fresh mulberry (*Morus*) leaves or artificial diets

Hui-Ling Dong[1,2], Sheng-Xiang Zhang[3], Hui Tao[1,2], Zhuo-Hua Chen[1,2], Xue Li[1,2], Jian-Feng Qiu[1,2], Wen-Zhao Cui [1,2], Yang-Hu Sima[1,2,4] , Wei-Zheng Cui[3], Shi-Qing Xu[1,2,4]

[1]School of Biology and Basic Medical Sciences, Medical College, Soochow University, Suzhou 215123, China. [2]Institute of Agricultural Biotechnology & Ecology (IABE), Soochow University, Suzhou 215123, China. [3]College of Forestry, Shandong Agricultural University, Taian Shandong 271018, China. [4]National Engineering Laboratory for Modern Silk (NEAER), Soochow University, Suzhou 215123, China. H. -L. D. and S. -X. Z. contributed equally to this work. Correspondence and requests for materials should be addressed to S.-Q.X. (email: szsqxu@suda.edu.cn) or W.-Z. C. (email: wzcui88@163.com).

Supplementary Information

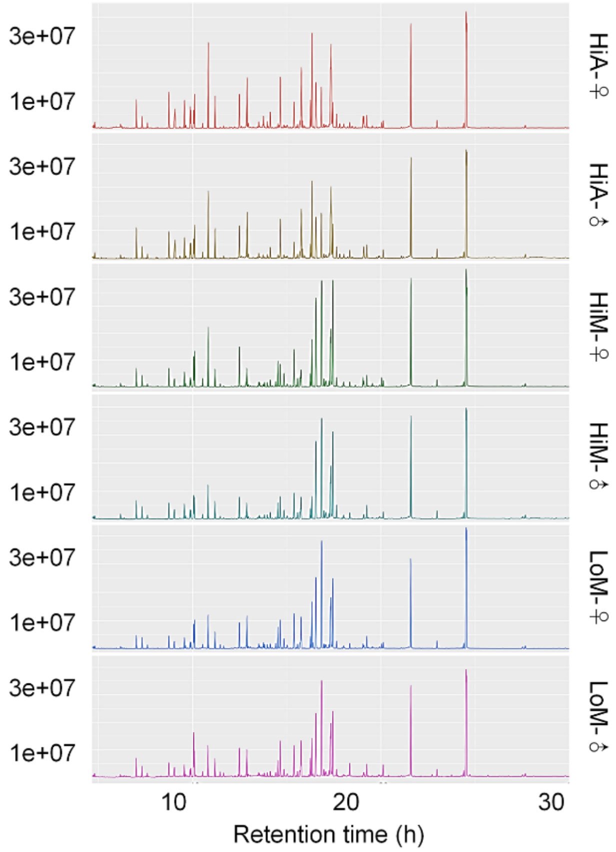

Figure S1 Total ion current GC-MS chromatogram. GC condition as: split injection with a split ratio of 20:1, injection volume 1 μl, the inlet, ion source and interface temperatures are 280℃, 250℃ and 150℃, respectively. Temperature programming were as follows: 70℃ for 2 min, then rise 10℃ per minute until 300℃ and keep 300℃ for 5 minutes. The total operating time was 30 min, the carrier gas was helium, and the carrier gas flow rate was 1 ml/min. MS condition as: electrospray ionization source, full scan mode, electron energy 70eV; quadrupole scan range 35 - 780 m/z.

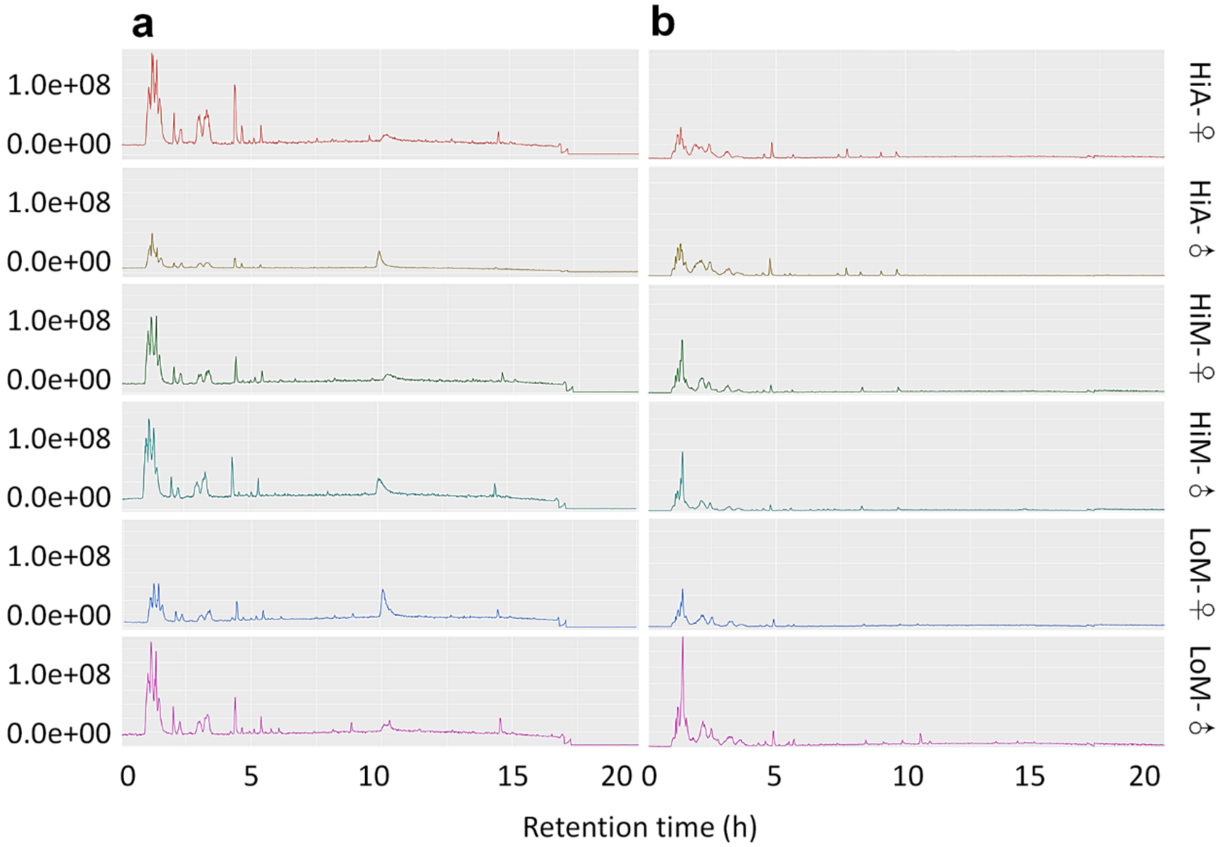

Figure S2 Total ion current LC-MS chromatograms. (a) Positive ion mode. (b) Negative ion mode. HPLC column temperature 35℃, injector temperature 8℃, injection volume 3 μl. The mobile phase is ultrapure water (A) / acetonitrile (B) (contain v/v = 0.1% formic acid). Flow rate 0. 3 ml/min, linear gradient elution (0 - 1min, 2% B; 1 - 11min, 2% - 50% B; 11 - 17min, 50% - 98% B; 17 -18 min, 98%; 18 - 19min, 98% - 2% B; 19 - 20min, 2% B). Leucine enkephalin as lock and sprey (0.4 ng/L, 0.1% formic acid CAN/H_2O=50/50). MS condition: mode capillary ionization voltage were 4.8 kV and 4.5 kV, respectively, Sheath gas 45, auxiliary gas 10, capillary temperature 325℃, scanning range positive ion 89 - 1000 m / z, negative ion 87 - 1000 m/z.

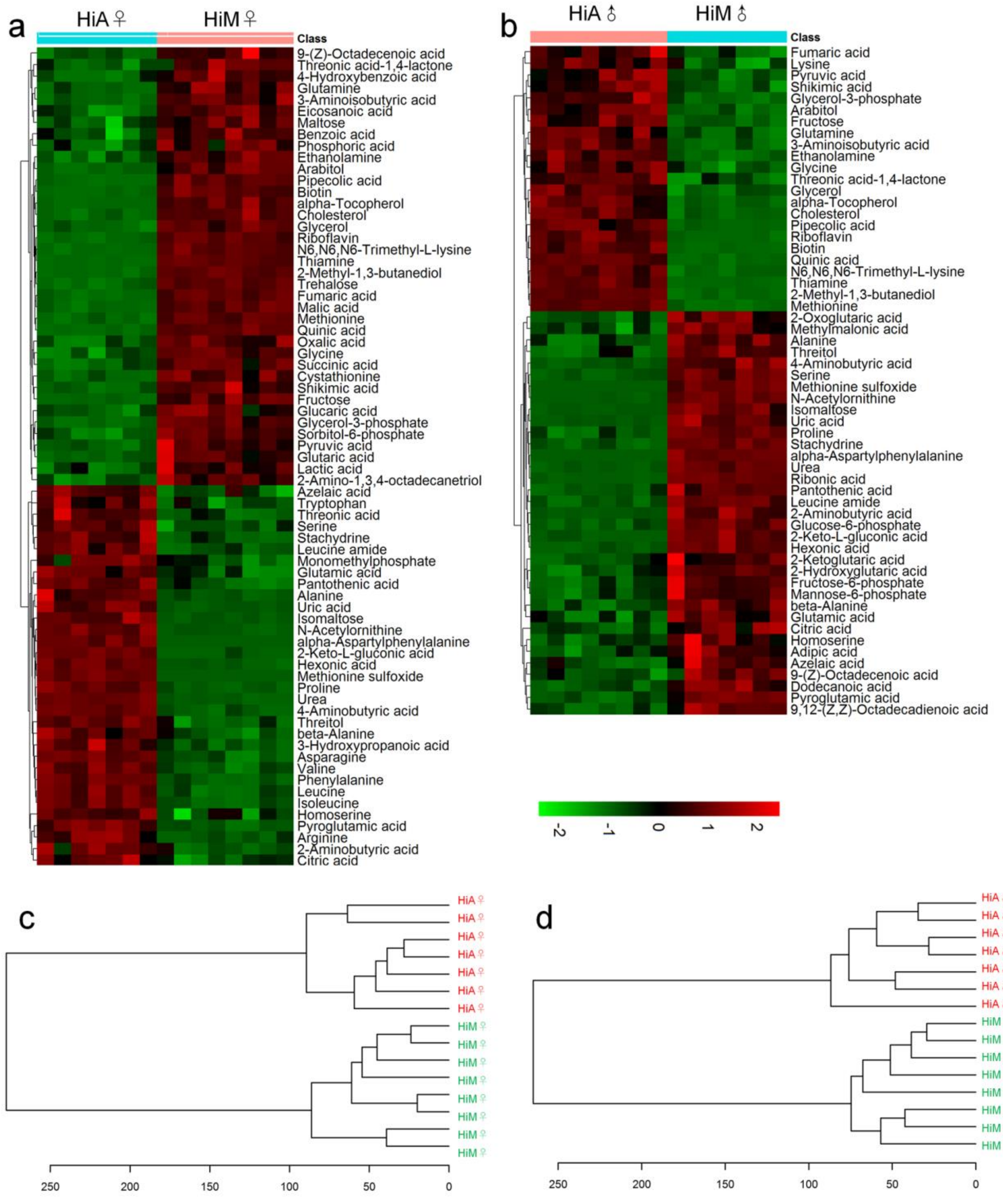

Figure S3 Differences metabolites heat map analysis (a ＆ b) and cluster analysis (c ＆ d). (a) and (c) are shown female, （b) and (d) represents the male. Heat map showing shades of metabolites in each group in an amount of height, the higher the amount of red, green represents the lower amount. Tree clustering was based on Euclidean clustering and the greatest distance method.

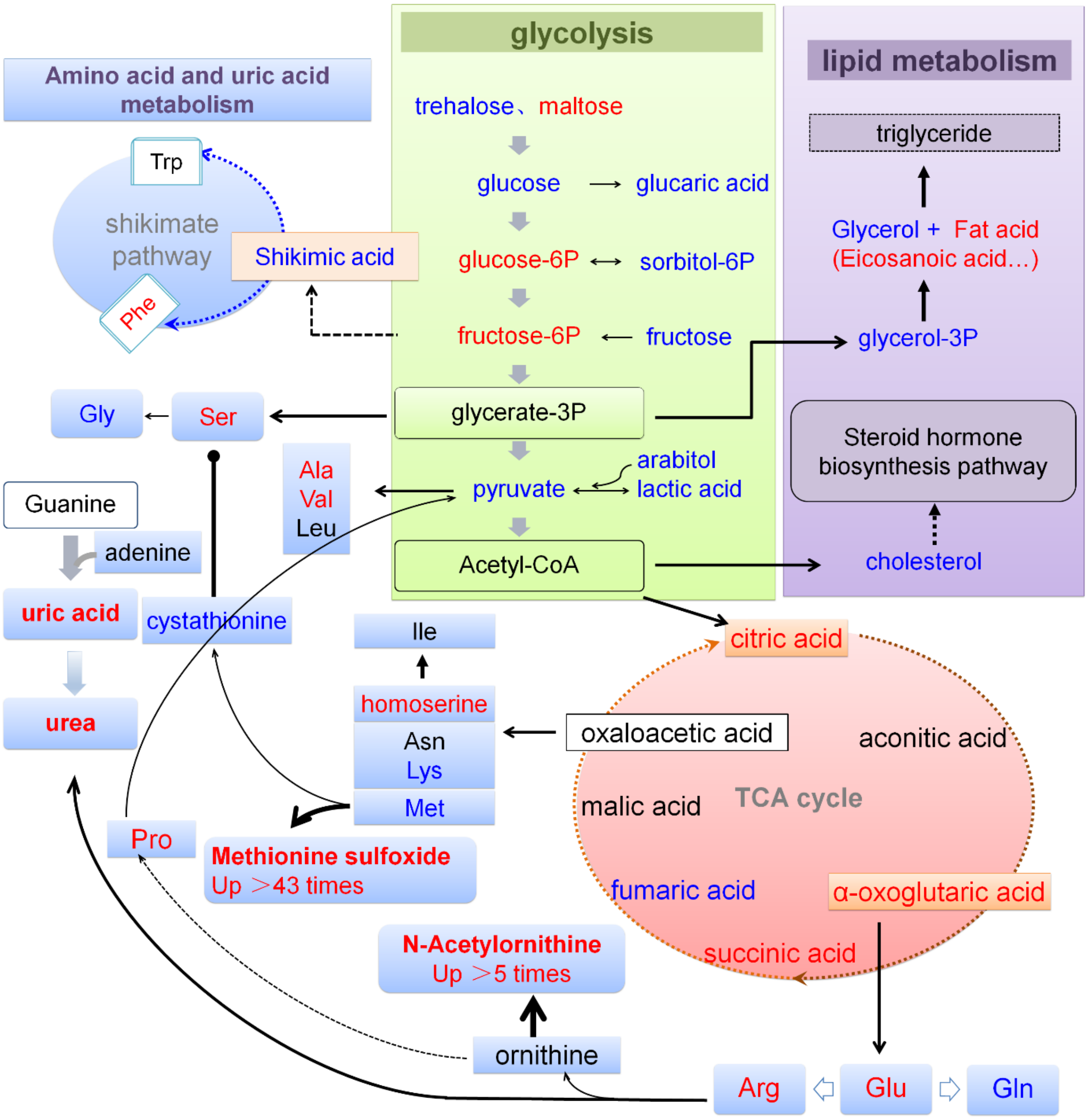

Figure S4 Summary of the primarily affected metabolic pathways in the hemolymph of male silkworms reared on the artificial diet. The pathways were simplified and organized based on Kyoto Encyclopedia of Genes and Genomes pathways (http://www.kegg.jp/). The data were collected from the metabolomic data of male larvae of the high feed intake strain reared on the artificial diet (HiA males). The font color represents the level of change in the silkworms reared on the artificial diet compared with that of the silkworms of the same strain reared on fresh mulberry leaves (HiM males); red indicates a higher level; blue indicates a lower level; and black indicates no significant change. Black font with a black frame indicates an unidentified metabolite.

Classification	Metabolites	Mz	Rt (mins)	Mean					
				HiM♀	HiM♂	LoM♀	LoM♂	HiA♀	HiA♂
**Amine**	Ethanolamine	174.1	9.5	0.90	1.28	0.98	1.06	0.67	0.92
	Leucine amide	3.0	131.1	0.15	0.10	0.23	0.17	0.38	0.55
**amino acid**	Alanine	116.1	7.0	52.67	55.61	50.26	61.89	63.50	76.32
	2-Aminobutyric acid	130.1	8.1	0.72	1.24	0.78	2.22	0.97	2.83
	Valine	144.1	8.8	50.49	52.67	41.58	47.16	70.88	57.17
	Leucine	158.1	9.6	45.01	53.60	38.98	42.77	63.66	50.23
	Isoleucine	158.1	9.9	24.20	28.78	20.84	22.31	39.97	28.94
	Proline	142.1	9.9	25.73	29.85	27.50	33.82	48.88	50.98
	Glycine	174.1	10.1	75.43	65.76	80.41	81.96	42.93	46.73
	Homoserine	103.0	10.7	0.02	0.02	0.02	0.02	0.03	0.04
	Serine	204.2	10.8	100.97	75.42	80.53	55.55	123.92	125.06
	beta-Alanine	248.1	11.7	1.35	1.64	4.21	5.48	1.83	2.50
	Methionine	176.1	12.8	9.42	7.24	6.91	6.20	3.46	3.17
	Pyroglutamic acid	156.1	12.9	58.01	67.59	110.16	109.73	126.96	126.63
	Hydroxyproline	140.1	13.0	0.97	1.10	0.94	1.00	0.97	1.21
	4-Aminobutyric acid	175.1	13.0	0.30	0.32	0.34	0.34	0.77	0.87
	Glutamic acid	246.1	14.0	1.36	1.38	1.98	1.31	2.41	2.12
	Phenylalanine	192.1	14.1	8.70	9.18	6.74	7.31	14.34	10.71
	Asparagine	116.1	14.7	21.19	30.10	33.13	47.44	35.72	30.92
	Lysine	174.1	17.3	36.28	35.78	37.71	36.04	32.70	28.91
	Prolinamide	1.2	147.1	26.77	25.19	34.35	22.39	28.60	26.49
	N6,N6,N6-Trimethyl-L-lysine	1.2	189.2	112.27	108.16	125.49	86.35	33.94	38.66
	Histidine	1.2	156.1	125.71	125.00	148.97	111.38	117.18	134.22
	Arginine	1.2	173.1	0.24	0.31	0.20	0.37	0.58	0.50
	Threonine	1.3	120.1	21.47	24.23	28.56	21.00	22.32	25.65
	N3,N4-Dimethyl-L-arginine	1.3	203.6	0.73	0.63	0.83	0.63	0.63	0.78
	2-Aminoadipic acid	1.4	162.1	2.15	2.64	3.45	3.55	2.12	2.47
	N-Acetylornithine	1.4	175.1	4.75	5.14	6.54	5.13	19.59	26.68
	4-Oxoproline	2.6	128.0	2.37	2.52	2.42	2.62	0.25	7.85
	Glutamine	3.5	145.1	2.98	2.53	0.04	0.12	0.85	0.56
	3-Aminoisobutyric acid	3.5	102.1	0.80	1.14	0.00	0.00	0.15	0.13
	Tryptophan	5.7	205.1	39.09	49.82	52.86	40.09	49.10	48.60
	α-Aspartylphenylalanine	5.9	281.1	0.08	0.10	0.15	0.14	2.91	3.93
**fatty acid**	Dodecanoic acid	117.0	14.3	0.07	0.06	0.05	0.06	0.08	0.11
	Tetradecanoic acid	132.0	16.4	0.04	0.04	0.03	0.03	0.04	0.06
	Hexonic acid	217.1	18.2	0.19	0.21	0.16	0.17	0.47	0.49
	Hexadecanoic acid	117.0	18.3	4.12	5.85	4.99	5.39	3.68	6.83
	Heptadecanoic acid	117.0	19.2	9.53	11.63	10.56	9.44	7.79	11.37
	Octadecanoic acid	117.0	20.1	3.08	4.33	3.65	3.88	2.68	5.75
	Eicosanoic acid	117.0	21.8	0.13	0.18	0.11	0.14	0.10	0.22
**nucleotides**	Uracil	241.1	10.5	0.60	0.60	0.43	0.59	0.50	0.75
	Thymine	255.1	11.3	0.05	0.07	0.05	0.06	0.06	0.07
	Adenine	2.1	136.1	0.15	0.24	0.14	0.10	0.11	0.29
	7-Methylguanine	2.7	166.1	0.35	0.38	0.43	0.31	0.31	0.35
**organic acid**	Pyruvic acid	174.0	6.2	6.22	7.95	3.53	4.15	3.84	5.42
	Lactic acid	117.1	6.4	1.33	1.53	1.14	1.04	0.92	1.30
	Glycolic acid	66.0	6.6	0.04	0.05	0.03	0.05	0.05	0.05
	Oxalic acid	66.0	7.5	0.02	0.03	0.02	0.04	0.01	0.03
	3-Hydroxypropanoic acid	177.1	7.6	0.13	0.24	0.13	0.43	0.19	0.31
	Malonic acid	45.0	8.5	0.04	0.05	0.04	0.03	0.03	0.04
	Benzoic acid	179.0	9.1	0.02	0.03	0.01	0.02	0.01	0.02
	Maleic acid	245.1	10.0	0.07	0.08	0.05	0.05	0.05	0.07
	Succinic acid	247.1	10.1	15.44	10.78	16.47	11.37	11.43	12.17
	Glyceric acid	292.1	10.4	0.14	0.15	0.14	0.14	0.16	0.16
	Fumaric acid	245.1	10.5	17.23	13.09	10.58	13.05	8.43	10.10
	Itaconic acid	43.1	10.6	0.20	0.16	0.11	0.12	0.10	0.18
	Pipecolic acid	156.1	10.8	4.30	6.71	6.18	6.40	0.75	0.88
	Glutaric acid	55.0	11.3	0.18	0.20	0.16	0.20	0.11	0.18
	Malic acid	233.1	12.5	20.39	14.89	15.02	16.78	13.71	14.19
	Adipic acid	111.0	12.6	0.17	0.16	0.14	0.16	0.14	0.22
	Tetronic acid	292.1	13.4	0.78	0.76	0.72	0.75	0.76	0.77
	2-Hydroxyglutaric acid	203.1	13.5	0.36	0.28	0.45	0.49	0.37	0.41
	2-Ketoglutaric acid	198.0	13.5	1.63	0.88	1.32	1.08	1.52	1.52
	4-Hydroxybenzoic acid	223.1	14.1	0.04	0.03	0.04	0.03	0.02	0.02
	Aconitic acid	375.1	15.5	0.38	0.25	0.28	0.32	0.06	0.13
	Ribonic acid	333.2	15.8	0.50	0.03	0.05	0.05	0.88	1.28

	Azelaic acid	55.1	15.9	0.03	0.06	0.04	0.05	0.05	0.11
	2-Keto-L-gluconic acid	292.1	15.9	0.02	0.02	0.05	0.01	0.60	0.59
	Shikimic acid	204.1	16.2	0.51	0.53	0.43	0.56	0.32	0.41
	Quinic acid	345.3	16.8	237.53	255.64	232.37	215.71	49.46	70.60
	Glucaric acid	333.1	18.4	0.25	0.26	0.25	0.28	0.21	0.23
	9,12-(Z,Z)-Octadecadienoic acid	81.1	19.9	0.08	0.16	0.08	0.14	0.20	0.59
	9-(Z)-Octadecenoic acid	117.0	19.9	0.62	0.91	0.63	0.65	0.48	1.35
	Urocanic acid	1.2	137.0	0.15	0.30	0.19	0.11	0.13	0.20
	Gluconic acid	1.3	195.1	13.55	18.00	14.92	16.24	14.37	14.12
	Threonic acid	1.3	135.0	0.62	0.59	0.60	0.85	1.44	1.63
	2-Oxoglutaric acid	1.6	145.0	30.94	14.54	23.48	39.46	34.13	30.24
	Glutaconic acid	2.2	111.0	51.21	66.70	53.82	40.93	69.26	74.92
	alpha-Hydroxyglutaric acid	2.2	147.0	2.21	3.15	2.81	3.83	1.95	2.72
	Uric acid	2.4	167.0	0.32	0.74	1.08	3.34	3.72	5.28
	Citric acid	2.5	191.0	129.67	85.52	106.21	141.91	180.79	125.11
	Methylmalonic acid	3.2	117.0	122.33	74.22	96.14	152.85	151.91	110.44
**Vitamins**	Pantothenic acid	103.1	18.0	1.56	1.48	1.31	1.37	2.19	2.38
	gamma-Tocopherol	223.1	26.0	0.14	0.15	0.15	0.14	0.11	0.19
	alpha-Tocopherol	237.1	27.3	0.34	0.39	0.27	0.25	0.05	0.09
	Thiamine	1.3	265.1	2.58	3.02	3.20	2.87	0.10	0.12
	Riboflavin	6.8	377.1	1.96	2.47	2.11	1.78	0.47	0.69
	Biotin	7.0	245.1	0.53	0.62	0.72	0.50	0.06	0.06
	Nicotinic acid	180.0	10.2	0.02	0.02	0.03	0.03	0.02	0.02
**others**	Urea	189.1	9.0	14.56	18.46	13.71	18.85	37.30	44.97
	1,3-Di-tert-butylbenzene	175.1	9.3	0.28	0.37	0.32	0.28	0.24	0.33
	Threonic acid-1,4-lactone	45.0	11.0	0.19	0.21	0.18	0.23	0.14	0.16
	Succinic anhydride	201.1	12.8	0.06	0.04	0.05	0.04	0.04	0.05
	beta-Sitosterol	129.1	29.8	0.48	0.55	0.63	0.56	0.41	0.50
	Choline	1.2	104.1	57.85	70.20	63.08	55.93	65.23	80.51
	Cholesterol	129.1	27.5	0.92	1.30	1.46	1.15	0.35	0.37
	Cystathionine	1.2	221.1	3.46	0.41	2.16	0.11	0.64	0.13
	Methionine sulfoxide	1.3	166.1	0.25	0.22	0.31	0.26	8.03	9.65
	Stachydrine	1.4	144.1	2.19	1.96	1.26	1.07	4.14	5.71
	Succinic semialdehyde	1.6	101.0	0.71	1.31	0.58	2.51	0.07	0.27
	Sepiapterin	2.5	238.1	6.44	5.86	6.55	4.85	6.24	5.99
	Esculin	5.7	339.1	0.07	0.18	0.07	0.09	0.07	0.05
	Betaine	8.3	118.1	21.21	29.04	20.52	15.85	28.88	35.89
	N,N'-Diphenylthiourea	12.9	229.2	0.14	0.15	0.15	0.09	0.09	0.15
**phosphoric acid**	Monomethylphosphate	241.0	8.2	0.09	0.09	0.07	0.08	0.12	0.12
	Phosphoric acid	299.1	9.6	6.49	6.06	6.17	5.63	5.83	6.34
	Glycerol-3-phosphate	299.1	15.7	8.20	9.02	6.42	7.51	3.44	5.23
	Fructose-6-phosphate	315.1	21.1	1.88	1.43	0.93	1.17	1.11	2.41
	Mannose-6-phosphate	387.2	21.1	0.93	0.64	0.46	0.54	0.51	1.06
	Glucose-6-phosphate	387.2	21.2	1.51	0.49	0.38	0.47	0.45	1.14
	Sorbitol-6-phosphate	299.1	21.5	1.48	1.51	1.00	1.04	0.78	1.23
**polyol**	Glycerol	205.1	9.6	1.13	1.39	1.11	1.21	0.66	0.82
	2-Methyl-1,3-butanediol	117.0	11.0	0.23	0.24	0.17	0.20	0.06	0.09
	Threitol	217.1	12.8	0.12	0.13	0.09	0.10	0.28	0.30
	Arabitol	217.1	15.2	1.50	1.27	1.04	1.12	0.86	0.65
	Ribitol	319.2	15.4	0.21	0.20	0.37	0.27	0.26	0.25
	Sorbitol	319.2	17.6	11.64	12.63	9.17	8.63	10.31	10.63
	myo-Inositol	217.1	19.1	8.09	4.58	4.81	4.00	12.01	7.08
	1-Monohexadecanoylglycerol	371.3	23.0	10.72	13.46	12.65	10.53	9.01	14.26
	Pyrogallol	3.5	125.0	0.16	0.51	0.27	0.39	0.20	0.76
	2-Amino-1,3,4-octadecanetriol	12.9	318.3	1.54	1.92	1.67	1.21	1.06	1.66
	2-Amino-1,3-octadecanediol	14.0	302.3	1.87	2.27	2.08	1.29	1.36	2.25
**sugar**	Fructose	103.1	17.0	6.46	6.64	4.69	6.31	2.54	2.86
	Galactose	133.0	17.2	0.85	0.08	0.21	0.06	0.12	0.11
	Glucose	103.0	17.3	1.53	0.77	0.78	0.83	0.36	0.50
	Trehalose	361.2	24.1	0.33	0.29	0.27	0.26	0.19	0.26
	Maltose	361.2	24.4	0.11	0.10	0.10	0.08	0.07	0.10
	Gentiobiose	362.1	25.0	0.10	0.07	0.06	0.06	0.06	0.07
	Isomaltose	361.2	25.6	0.08	0.05	0.05	0.03	0.47	0.37
	Sucrose	1.3	387.1	47.76	47.05	41.89	34.16	47.20	55.02

Table S1 Differences in the metabolites examined by LC/GC-MS. Mz, quantitative ion of metabolites. Rt (mins), retention time (minutes). Mean, Repeat measurement of biological samples are averaged.

Metabolites	Mz	Rt (mins)	Mean		S.D.		VIP	pvalue	qvalue	log_2FC
			HiA♀	HiM♀+LoM♀	HiA♀	HiM♀+LoM♀				
**Thiamine**	265.1	1.3	0.10	2.89	0.01	0.37	1.45	0.00	0.00	-4.79
**Biotin**	245.1	7.0	0.06	0.62	0.01	0.13	1.38	0.00	0.00	-3.38
**Pipecolic acid**	156.1	10.8	0.75	5.24	0.12	1.16	1.35	0.00	0.00	-2.80
**alpha-Tocopherol**	237.1	27.3	0.05	0.31	0.00	0.06	1.35	0.00	0.00	-2.52
**Quinic acid**	345.3	16.8	49.46	234.95	4.82	17.11	1.47	0.00	0.00	-2.25
**Cystathionine**	221.1	1.2	0.64	2.81	0.23	0.93	1.18	0.00	0.00	-2.13
**Riboflavin**	377.1	6.8	0.47	2.03	0.06	0.22	1.44	0.00	0.00	-2.10
**N6,N6,N6-Trimethyl-L-lysine**	189.2	1.2	33.94	118.88	2.37	10.35	1.45	0.00	0.00	-1.81
**Cholesterol**	129.1	27.5	0.35	1.19	0.04	0.33	1.23	0.00	0.00	-1.76
**2-Methyl-1,3-butanediol**	117.0	11.0	0.06	0.20	0.01	0.03	1.36	0.00	0.00	-1.69
**Methionine**	176.1	12.8	3.46	8.16	0.35	1.39	1.32	0.00	0.00	-1.24
**Oxalic acid**	66.0	7.5	0.01	0.02	0.00	0.00	1.30	0.00	0.00	-1.21
**Fructose**	103.1	17.0	2.54	5.58	0.11	1.13	1.24	0.00	0.00	-1.13
**Glycerol-3-phosphate**	299.1	15.7	3.44	7.31	0.28	1.28	1.28	0.00	0.00	-1.09
**4-Hydroxybenzoic acid**	223.1	14.1	0.02	0.04	0.00	0.00	1.33	0.00	0.00	-0.96
**Glycine**	174.1	10.1	42.93	77.92	6.96	8.40	1.34	0.00	0.00	-0.86
**Glycerol**	205.1	9.6	0.66	1.12	0.04	0.07	1.42	0.00	0.00	-0.76
**N,N-Diphenylthiourea**	229.2	12.9	0.09	0.15	0.02	0.03	1.05	0.00	0.01	-0.66
**Glutaric acid**	55.0	11.3	0.11	0.17	0.00	0.02	1.24	0.00	0.00	-0.64
**Trehalose**	361.2	24.1	0.19	0.30	0.01	0.03	1.30	0.00	0.00	-0.61
**2-Amino-1,3,4-octadecanetriol**	318.3	12.9	1.06	1.60	0.10	0.24	1.16	0.00	0.00	-0.60
**2-Amino-1,3-octadecanediol**	302.3	14.0	1.36	1.97	0.25	0.30	1.07	0.00	0.01	-0.54
**Shikimic acid**	204.1	16.2	0.32	0.47	0.02	0.07	1.13	0.00	0.00	-0.53
**Ethanolamine**	174.1	9.5	0.67	0.94	0.04	0.08	1.30	0.00	0.00	-0.48
**Succinic acid**	247.1	10.1	11.43	15.95	0.60	2.14	1.14	0.00	0.00	-0.48
**Maltose**	361.2	24.4	0.07	0.10	0.01	0.01	1.12	0.00	0.00	-0.45
**Threonic acid-1,4-lactone**	45.0	11.0	0.14	0.19	0.01	0.02	1.18	0.00	0.00	-0.44
**9-(Z)-Octadecenoic acid**	117.0	19.9	0.48	0.62	0.02	0.07	1.16	0.00	0.00	-0.39
**Eicosanoic acid**	117.0	21.8	0.10	0.12	0.01	0.01	1.07	0.00	0.01	-0.32
**Glucaric acid**	333.1	18.4	0.21	0.25	0.01	0.02	1.18	0.00	0.00	-0.24
**Lysine**	174.1	17.3	32.70	36.99	2.16	1.83	1.09	0.00	0.01	-0.18
**Alanine**	116.1	7.0	63.50	51.46	3.51	2.63	1.33	0.00	0.00	0.30
**2-Aminobutyric acid**	130.1	8.1	0.97	0.75	0.11	0.07	1.17	0.00	0.00	0.37
**Serine**	204.2	10.8	123.92	90.75	5.57	11.80	1.25	0.00	0.00	0.45
**Methylmalonic acid**	117.0	3.2	151.91	109.23	22.35	17.02	1.10	0.00	0.00	0.48
**3-Hydroxypropanoic acid**	177.1	7.6	0.19	0.13	0.02	0.01	1.39	0.00	0.00	0.55
**Monomethylphosphate**	241.0	8.2	0.12	0.08	0.01	0.01	1.23	0.00	0.00	0.56
**Glycolic acid**	66.0	6.6	0.05	0.03	0.01	0.01	1.09	0.00	0.00	0.56
**Leucine**	158.1	9.6	63.66	41.99	3.16	3.87	1.40	0.00	0.00	0.60
**Pantothenic acid**	103.1	18.0	2.19	1.44	0.10	0.19	1.34	0.00	0.00	0.61
**Citric acid**	191.0	2.5	180.79	117.94	20.91	20.53	1.23	0.00	0.00	0.62
**Valine**	144.1	8.8	70.88	46.03	3.66	5.29	1.38	0.00	0.00	0.62
**Azelaic acid**	55.1	15.9	0.05	0.03	0.01	0.01	1.16	0.00	0.00	0.69
**Homoserine**	103.0	10.7	0.03	0.02	0.00	0.01	1.08	0.00	0.01	0.74
**Isoleucine**	158.1	9.9	39.97	22.52	2.28	2.21	1.44	0.00	0.00	0.83
**Proline**	142.1	9.9	48.88	26.61	2.89	2.05	1.45	0.00	0.00	0.88
**Phenylalanine**	192.1	14.1	14.34	7.72	0.59	1.10	1.42	0.00	0.00	0.89
**Leucine amide**	131.1	3.0	0.38	0.19	0.06	0.06	1.25	0.00	0.00	0.99
**Threonic acid**	135.0	1.3	1.44	0.61	0.27	0.13	1.35	0.00	0.00	1.24
**Stachydrine**	144.1	1.4	4.14	1.72	0.51	0.56	1.34	0.00	0.00	1.26
**4-Aminobutyric acid**	175.1	13.0	0.77	0.32	0.04	0.07	1.43	0.00	0.00	1.28
**9,12-(Z,Z)-Octadecadienoic acid**	81.1	19.9	0.20	0.08	0.11	0.03	1.01	0.00	0.02	1.31
**Threitol**	217.1	12.8	0.28	0.11	0.02	0.04	1.35	0.00	0.00	1.40
**Arginine**	173.1	1.2	0.58	0.22	0.12	0.04	1.38	0.00	0.00	1.40
**Urea**	189.1	9.0	37.30	14.13	1.35	1.08	1.48	0.00	0.00	1.40
**Hexonic acid**	217.1	18.2	0.47	0.17	0.03	0.02	1.47	0.00	0.00	1.43
**N-Acetylornithine**	175.1	1.4	19.59	5.64	2.07	1.09	1.45	0.00	0.00	1.80
**Uric acid**	167.0	2.4	3.72	0.70	0.85	0.45	1.38	0.00	0.00	2.41
**Isomaltose**	361.2	25.6	0.47	0.06	0.08	0.03	1.44	0.00	0.00	2.88
**2-Keto-L-gluconic acid**	292.1	15.9	0.60	0.04	0.04	0.04	1.47	0.00	0.00	4.04
**alpha-Aspartylphenylalanine**	281.1	5.9	2.91	0.12	0.33	0.04	1.47	0.00	0.00	4.66
**Methionine sulfoxide**	166.1	1.3	8.03	0.28	0.60	0.05	1.48	0.00	0.00	4.85

Table S2 Metabolites in hemolymph of female silkworm larvae examined by LC/GC-MS. RI, retention index. Mz, quantitative ion of metabolites. Rt (mins), retention time (minutes). Mean, Repeat measurement of

biological samples are averaged. S.D., standard variance. pvalue, *t*-test significance. qvalue, Also known as false discovery rate (FDR), Which is false-positive correction of t-test significance. $\log_2$FC, logarithm of (HiM♀+LoM♀) /HiA♀ fold change ($\log_2$FC >0 means (HiM♀+LoM♀) < HiA; $\log_2$FC <0 means (HiM♀+LoM♀) >HiA). VIP, Partial least squares discrimination analysis (PLS-DA) first principal component variable importance in projection.

Metabolites	mz	Rt (mins)	mean		S.D.		VIP	pvalue	qvalue	$\log_2$FC
			HiA♂	HiM♂+LoM♂	HiA♂	HiM♂+LoM♂				
**Thiamine**	265.1	1.3	0.12	2.94	0.04	0.43	1.53	0.00	0.00	-4.65
**Biotin**	245.1	7.0	0.06	0.56	0.01	0.11	1.48	0.00	0.00	-3.34
**Pipecolic acid**	156.1	10.8	0.88	6.55	0.21	1.33	1.47	0.00	0.00	-2.90
**alpha-Tocopherol**	237.1	27.3	0.09	0.32	0.02	0.10	1.29	0.00	0.00	-1.89
**Quinic acid**	345.3	16.8	70.60	235.68	7.21	27.47	1.52	0.00	0.00	-1.74
**Cholesterol**	129.1	27.5	0.37	1.23	0.08	0.23	1.43	0.00	0.00	-1.72
**Riboflavin**	377.1	6.8	0.69	2.12	0.06	0.46	1.38	0.00	0.00	-1.63
**N6,N6,N6-Trimethyl-L-lysine**	189.2	1.2	38.66	97.25	4.51	15.89	1.43	0.00	0.00	-1.33
**2-Methyl-1,3-butanediol**	117.0	11.0	0.09	0.22	0.01	0.02	1.52	0.00	0.00	-1.30
**Fructose**	103.1	17.0	2.86	6.47	0.38	0.97	1.43	0.00	0.00	-1.18
**Methionine**	176.1	12.8	3.17	6.72	0.21	0.70	1.50	0.00	0.00	-1.08
**Arabitol**	217.1	15.2	0.65	1.20	0.07	0.18	1.37	0.00	0.00	-0.87
**Glucose**	103.0	17.3	0.50	0.80	0.07	0.15	1.17	0.00	0.00	-0.67
**Glycerol-3-phosphate**	299.1	15.7	5.23	8.26	0.46	1.22	1.29	0.00	0.00	-0.66
**Glycerol**	205.1	9.6	0.82	1.30	0.09	0.14	1.39	0.00	0.00	-0.66
**Glycine**	174.1	10.1	46.73	73.86	4.80	12.85	1.21	0.00	0.00	-0.66
**4-Hydroxybenzoic acid**	223.1	14.1	0.02	0.03	0.00	0.01	1.05	0.00	0.04	-0.62
**Threonic acid-1,4-lactone**	45.0	11.0	0.16	0.22	0.01	0.02	1.39	0.00	0.00	-0.50
**Shikimic acid**	204.1	16.2	0.41	0.55	0.03	0.08	1.12	0.00	0.01	-0.42
**Fumaric acid**	245.1	10.5	10.10	13.07	0.80	1.44	1.18	0.00	0.00	-0.37
**Ethanolamine**	174.1	9.5	0.92	1.17	0.03	0.14	1.14	0.00	0.01	-0.35
**Lysine**	174.1	17.3	28.91	35.91	3.01	2.46	1.25	0.00	0.00	-0.31
**Glucaric acid**	333.1	18.4	0.23	0.27	0.01	0.02	1.06	0.00	0.04	-0.22
**Choline**	104.1	1.2	80.51	63.07	6.44	10.20	1.07	0.00	0.04	0.35
**Phenylalanine**	192.1	14.1	10.71	8.25	0.86	1.13	1.18	0.00	0.00	0.38
**Alanine**	116.1	7.0	76.32	58.75	5.04	5.21	1.36	0.00	0.00	0.38
**Adipic acid**	111.0	12.6	0.22	0.16	0.03	0.02	1.29	0.00	0.00	0.46
**Monomethylphosphate**	241.0	8.2	0.12	0.08	0.02	0.01	1.14	0.00	0.01	0.48
**2-Ketoglutaric acid**	198.0	13.5	1.52	0.98	0.32	0.15	1.23	0.00	0.00	0.63
**Glutamic acid**	246.1	14.0	2.12	1.35	0.28	0.24	1.32	0.00	0.00	0.66
**Proline**	142.1	9.9	50.98	31.83	2.23	3.39	1.51	0.00	0.00	0.68
**2-Aminobutyric acid**	130.1	8.1	2.83	1.73	0.47	0.55	1.12	0.00	0.01	0.71
**myo-Inositol**	217.1	19.1	7.08	4.29	1.24	1.11	1.21	0.00	0.00	0.72
**Pantothenic acid**	103.1	18.0	2.38	1.43	0.24	0.12	1.50	0.00	0.00	0.74
**9-(Z)-Octadecenoic acid**	117.0	19.9	1.35	0.78	0.23	0.30	1.11	0.00	0.02	0.79
**Mannose-6-phosphate**	387.2	21.1	1.06	0.59	0.16	0.11	1.40	0.00	0.00	0.85
**Dodecanoic acid**	117.0	14.3	0.11	0.06	0.02	0.01	1.36	0.00	0.00	0.89
**Fructose-6-phosphate**	315.1	21.1	2.41	1.30	0.35	0.25	1.41	0.00	0.00	0.89
**Homoserine**	103.0	10.7	0.04	0.02	0.01	0.01	1.22	0.00	0.00	0.93
**Serine**	204.2	10.8	125.06	65.48	8.44	11.10	1.49	0.00	0.00	0.93
**Azelaic acid**	55.1	15.9	0.11	0.05	0.02	0.01	1.34	0.00	0.00	1.04
**Threonic acid**	135.0	1.3	1.63	0.72	0.86	0.23	1.05	0.00	0.04	1.19

**Glucose-6-phosphate**	387.2	21.2	1.14	0.48	0.11	0.07	1.54	0.00	0.00	1.25
**Urea**	189.1	9.0	44.97	18.66	2.83	2.00	1.56	0.00	0.00	1.27
**Hexonic acid**	217.1	18.2	0.49	0.19	0.04	0.02	1.55	0.00	0.00	1.33
**Uric acid**	167.0	2.4	5.28	2.04	1.11	1.56	1.16	0.00	0.01	1.37
**4-Aminobutyric acid**	175.1	13.0	0.87	0.33	0.13	0.04	1.52	0.00	0.00	1.39
**Threitol**	217.1	12.8	0.30	0.11	0.04	0.05	1.43	0.00	0.00	1.41
**Stachydrine**	144.1	1.4	5.71	1.51	0.33	0.53	1.54	0.00	0.00	1.92
**9,12-(Z,Z)-Octadecadienoic acid**	81.1	19.9	0.59	0.15	0.14	0.16	1.29	0.00	0.00	1.99
**Leucine amide**	131.1	3.0	0.55	0.13	0.07	0.05	1.53	0.00	0.00	2.05
**N-Acetylornithine**	175.1	1.4	26.68	5.14	1.89	0.84	1.58	0.00	0.00	2.38
**Isomaltose**	361.2	25.6	0.37	0.04	0.07	0.02	1.54	0.00	0.00	3.15
**Ribonic acid**	333.2	15.8	1.28	0.04	0.08	0.02	1.58	0.00	0.00	4.92
**alpha-Aspartylphenylalanine**	281.1	5.9	3.93	0.12	0.39	0.03	1.58	0.00	0.00	5.02
**Methionine sulfoxide**	166.1	1.3	9.65	0.24	1.15	0.04	1.57	0.00	0.00	5.31
**2-Keto-L-gluconic acid**	292.1	15.9	0.59	0.01	0.10	0.02	1.56	0.00	0.00	5.46

Table S3 Metabolites in hemolymph of male silkworm larvae examined by LC/GC-MS. RI, retention index. Mz, quantitative ion of metabolites. Rt (mins), retention time (minutes). Mean, Repeat measurement of biological samples are averaged. S.D., standard variance. pvalue, *t*-test significance. qvalue, Also known as false discovery rate (FDR), Which is false-positive correction of t-test significance. log_2FC, logarithm of (HiM♂+LoM♂) /HiA♂ fold change (log_2FC >0 means (HiM♂+LoM♂) < HiA; log2FC <0 means (HiM♂+LoM♂) >HiA). VIP, Partial least squares discrimination analysis (PLS-DA) first principal component variable importance in projection.

**Metabolites**	**Mean**			**HiM♀ / HiA♀**			**LoM♀ / HiA♀**		
	HiM♀	LoM♀	HiA♀	VIP	pvalue	log_2FC	VIP	pvalue	log_2FC
**beta-Alanine**	1.35	4.21	1.83	1.16	0.00	-0.45	1.25	0.00	1.20
**Threonine**	21.47	28.56	22.32	0.24	0.52	-0.06	1.09	0.00	0.36
**Tryptophan**	39.09	52.86	49.10	1.07	0.00	-0.33	0.44	0.22	0.11
**3-Aminoisobutyric acid**	0.80	0.00	0.15	1.24	0.00	2.44	1.20	0.00	-∞
**Glutamine**	2.98	0.04	0.85	1.19	0.00	1.81	1.22	0.00	-4.39
**Pyruvic acid**	6.22	3.53	3.84	1.18	0.00	0.70	0.58	0.10	-0.12
**Benzoic acid**	0.02	0.01	0.01	1.13	0.00	0.65	0.26	0.47	-0.17

Table S4 Differences in the metabolites between the female silkworm larvae reared on fresh mulberry leaves and on artificial diet. Mean, Repeat measurement of biological samples are averaged.. pvalue, *t*-test significance. log_2FC, logarithm of (HiM♀ or LoM♀) /HiA♀ fold change. VIP, Partial least squares discrimination analysis (PLS-DA) first principal component variable importance in projection.

Metabolites	Mean			HiA♂ / HiM♂			HiA♂ / LoM♂		
	HiA♂	HiM♂	LoM♂	VIP	pvalue	log_2FC	VIP	pvalue	log_2FC
**3-Aminoisobutyric acid**	0.13	1.14	0.00	1.36	0.00	-3.19	0.88	0.01	6.29
**Glutamine**	0.56	2.53	0.12	1.28	0.00	-2.16	0.96	0.00	2.18
**Urocanic acid**	0.20	0.30	0.11	1.02	0.00	-0.61	0.88	0.01	0.83
**Pyruvic acid**	5.42	7.95	4.15	1.18	0.00	-0.55	1.21	0.00	0.39
**N,N-Diphenylthiourea**	0.15	0.15	0.09	0.09	0.82	-0.02	1.10	0.00	0.80
**Sorbitol-6-phosphate**	1.23	1.51	1.04	1.07	0.00	-0.29	0.76	0.03	0.24
**2-Amino-1,3-octadecanediol**	2.25	2.27	1.29	0.03	0.95	-0.01	1.13	0.00	0.80
**beta-Alanine**	2.50	1.64	5.48	1.31	0.00	0.60	1.29	0.00	-1.13
**2-Hydroxyglutaric acid**	0.41	0.28	0.49	1.27	0.00	0.54	0.89	0.01	-0.27
**3-Hydroxypropanoic acid**	0.31	0.24	0.43	1.01	0.00	0.40	1.02	0.00	-0.45
**Methylmalonic acid**	110.44	74.22	152.85	1.23	0.00	0.57	0.97	0.00	-0.47
**2-Oxoglutaric acid**	30.24	14.54	39.46	1.25	0.00	1.06	0.60	0.10	-0.38
**Citric acid**	125.11	85.52	141.91	1.17	0.00	0.55	0.47	0.22	-0.18
**Asparagine**	30.92	30.10	47.44	0.18	0.66	0.04	1.12	0.00	-0.62

Table S5 Differences in the metabolites between the male silkworm larvae reared on fresh mulberry leaves and on artificial diet. Mean, Repeat measurement of biological samples are averaged.. pvalue, *t*-test significance. log_2FC, logarithm of (HiM♂ or LoM♂) /HiA♂ fold change. VIP, Partial least squares discrimination analysis (PLS-DA) first principal component variable importance in projection.

**Year**	**0**	**1**	**2**	**3**	**4**	**5**	**6**	**7**
**Select generation**	S_0	S_2	S_4	S_6	S_8	S_{10}	S_{12}	S_{14}
**PST of Hi (%)**	48.2	69.1	82.7	89.5	93.9	96.6	100	100
**PST of Lo (%)**	48.2	40.4	31.9	26.7	17.4	9.2	2.3	0.80

Table S6 Effect of disruptive selection on the ingestive behavior of Jingsong B silkworm varieties to artificial diet. S_0, the generation of mother strain. S_2-S_{14}, the second to the 14th select generation. The starting material was a domesticated silkworm variety, B. mori Jingsong B (Chinese system). Using a combination of batch selection and individual selection, a continuous, we conducted a multi-generational disruptive selection of the ingestive behavior of silkworm larvae to an artificial diet. We judged feeding performance by the percentage of setae dispersion after feeding 48h (PST). The body of newly-hatched silkworm larvae is black. The body would light in color after normal eating mulberry leaf or artificial diet more than 48 hours, meantime the bulged setae tubercle on cuticle would flattened. If the larvae can't eat normally, they will retarded growth and the bulged setae tubercle on cuticle cannot be flattened. After 7 years of continuous selection of 14 generations, we obtained two strains, one of which almost 100% of the larvae ingested the artificial diet, while the other almost 100% of the larvae did not ingested the artificial diet and starved to death during their newly hatched larval stage. They are named as high ingestive habit stain and lower ingestive habit stain, and abbreviated to Hi and Lo in this study, respectively. The data repeat 10 groups.
